# Guideline appraisal with AGREE II: online survey of the potential influence of AGREE II items on overall assessment of guideline quality and recommendation for use

**DOI:** 10.1186/s12913-018-2954-8

**Published:** 2018-02-27

**Authors:** Wiebke Hoffmann-Eßer, Ulrich Siering, Edmund A. M. Neugebauer, Anne Catharina Brockhaus, Natalie McGauran, Michaela Eikermann

**Affiliations:** 1 Institute for Quality and Efficiency in Health Care (IQWiG), Im Mediapark 8, 50670 Cologne, Germany; 2 Institute for Research in Operative Medicine (IFOM), University of Witten/Herdecke, Campus Cologne, Cologne, Germany; 3 Senior Professor for Health Services Research, University of Witten/Herdecke, Alfred-Herrhausen-Straße 50, 58448 Witten, Germany; 4 Medical Advisory Service of the German Social Health Insurance (MDS), Theodor-Althoff-Straße 47, 45133 Essen, Germany

**Keywords:** Clinical practice guidelines, Methodological guideline appraisal, Methodological quality, Systematic reviews

## Abstract

**Background:**

The AGREE II instrument is the most commonly used guideline appraisal tool. It includes 23 appraisal criteria (items) organized within six domains. AGREE II also includes two overall assessments (overall guideline quality, recommendation for use). Our aim was to investigate how strongly the 23 AGREE II items influence the two overall assessments.

**Methods:**

An online survey of authors of publications on guideline appraisals with AGREE II and guideline users from a German scientific network was conducted between 10th February 2015 and 30th March 2015. Participants were asked to rate the influence of the AGREE II items on a Likert scale (0 = no influence to 5 = very strong influence). The frequencies of responses and their dispersion were presented descriptively.

**Results:**

Fifty-eight of the 376 persons contacted (15.4%) participated in the survey and the data of the 51 respondents with prior knowledge of AGREE II were analysed. Items 7–12 of Domain 3 (rigour of development) and both items of Domain 6 (editorial independence) had the strongest influence on the two overall assessments. In addition, Items 15–17 (clarity of presentation) had a strong influence on the recommendation for use. Great variations were shown for the other items. The main limitation of the survey is the low response rate.

**Conclusions:**

In guideline appraisals using AGREE II, items representing rigour of guideline development and editorial independence seem to have the strongest influence on the two overall assessments. In order to ensure a transparent approach to reaching the overall assessments, we suggest the inclusion of a recommendation in the AGREE II user manual on how to consider item and domain scores. For instance, the manual could include an a-priori weighting of those items and domains that should have the strongest influence on the two overall assessments. The relevance of these assessments within AGREE II could thereby be further specified.

**Electronic supplementary material:**

The online version of this article (10.1186/s12913-018-2954-8) contains supplementary material, which is available to authorized users.

## Background

According to the definition of the US Institute of Medicine (IOM), “clinical practice guidelines are statements that include recommendations intended to optimize patient care that are informed by a systematic review of evidence and an assessment of the benefits and harms of alternative care options” [1, 2]. Various studies have shown that guidelines can improve health care [3–9]; however, their quality is variable and often unsatisfactory [10–14]. In order to be able to use guidelines as a reliable basis for decision-making, their quality, i.e. their methodological rigour and transparency, needs to be ensured. Guideline appraisal tools are applied for this purpose.

In 2003, an international group of guideline developers and researchers developed the Appraisal of Guidelines for Research & Evaluation (AGREE) instrument [15]. The revised version, AGREE II [16], was published in 2009 and is currently the most commonly applied and comprehensively validated guideline appraisal tool worldwide [17–19]. It consists of 23 appraisal criteria (items) organized into six domains (Table 1), each of which “captures a unique dimension of guideline quality” [16]. The items within each domain are rated on a seven-point scale (“strongly disagree” to “strongly agree”).

**Table 1:** Items and domains of the AGREE II instrument^a^

Item	Content	Domain
1	The overall objective(s) of the guideline is (are) specifically described.	Scope and Purpose
2	The health question(s) covered by the guideline is (are) specifically described.	
3	The population (patients, public, etc.) to whom the guideline is meant to apply is specifically described.	
4	The guideline development group includes individuals from all relevant professional groups.	Stakeholder Involvement
5	The views and preferences of the target population (patients, public, etc.) have been sought.	
6	The target users of the guideline are clearly defined.	
7	Systematic methods were used to search for evidence.	Rigour of Development
8	The criteria for selecting the evidence are clearly described.	
9	The strengths and limitations of the body of evidence are clearly described.	
10	The methods for formulating the recommendations are clearly described.	
11	The health benefits, side effects, and risks have been considered in formulating the recommendations.	
12	There is an explicit link between the recommendations and the supporting evidence.	
13	The guideline has been externally reviewed by experts prior to its publication.	
14	A procedure for updating the guideline is provided.	
15	The recommendations are specific and unambiguous.	Clarity of Presentation
16	The different options for management of the condition or health issue are clearly presented.	
17	Key recommendations are easily identifiable.	
18	The guideline describes facilitators and barriers to its application.	Applicability
19	The guideline provides advice and/or tools on how the recommendations can be put into practice.	
20	The potential resource implications of applying the recommendations have been considered.	
21	The guideline presents monitoring and/or auditing criteria.	
22	The views of the funding body have not influenced the content of the guideline.	Editorial Independence
23	Competing interests of guideline development group members have been recorded and addressed.	

^a^Extracted from [16]

In addition, AGREE II includes two global rating items (overall assessments). In the first assessment, the overall guideline quality is rated on a seven-point scale (“lowest possible quality” to “highest possible quality”). In the second assessment, a recommendation is provided on whether to use the guideline or not (“yes”, “yes with modifications”, “no”). Both assessments should consider the items evaluated beforehand and the resulting domain scores, but should not be calculated from them: it is explicitly noted that the “six domain scores are independent and should not be aggregated into a single quality score” [16]). Beyond this information, AGREE II does not provide a specific approach to reaching the two overall assessments. The lack of operationalization for the conduct of the two overall assessments results in inconsistent approaches by guideline users, leading to subjective assessments [20–24].

In a recently published systematic review based on publications reporting guideline appraisals with AGREE II, we investigated how often AGREE II users conducted the two overall assessments and to what extent the six domain scores influenced these assessments [25]. We found that the two overall assessments were underreported by guideline assessors. Domains 3 (rigour of development) and 5 (applicability) had the strongest influence on the results of the two overall assessments, while the other domains had a varying influence.

Despite the deficits described above, the two overall assessments of AGREE II provide important information on whether a user can regard a guideline to be reliable, for example, as a basis for guideline development [26] or for application in clinical practice.

The above systematic review only investigated how strongly the six domains (and not the individual items) influenced the two overall assessments and was based on the published literature. The present analysis is an extension of the systematic review and aimed to provide a more detailed examination with a more practical orientation: on the basis of a survey of guideline users we investigated how strongly the 23 individual AGREE II items influenced the two overall assessments.

## Methods

### Conduct of the survey

We performed a systematic search to identify publications reporting results of guideline appraisals with AGREE II. We then asked the corresponding authors of these publications, as well as a group of further guideline users (all members of the Guidelines Section of the German Network for Evidence-based Medicine, DNEbM), to participate in an online survey conducted via Survey Monkey between 10 February and 30 March 2015. The link to the survey was included in the e-mail. The DNEbM members received a version including an introductory text and explanations in German plus the original AGREE II items in English; the corresponding authors of publications received a completely English version (see Additional file 1). A reminder e-mail was sent two weeks before the end of the deadline.

The focus of the survey was on the assessment of the strength of the potential influence of the AGREE II items on the two overall assessments (overall guideline quality and recommendation for use). For each of the 23 AGREE II items, respondents rated the strength of the influence on a Likert scale (0 = no influence to 5 = very strong influence). In addition, respondents were asked to provide information on characteristics such as their profession, knowledge of AGREE II, practical experience with the original AGREE instrument (AGREE I) or AGREE II, the purpose of guideline appraisals with AGREE I or II, and any prior involvement in guideline development. Furthermore, the survey contained an open question on which items respondents used in the overall assessment of guideline quality.

### Data analysis

We analysed the combined results of the German and English versions of the survey using SPSS (PASW Statistics 18 [frequencies]) and SAS.

We presented the results descriptively; the respondents’ characteristics were presented in a table; the respondents’ evaluation of the influence of the AGREE II items on the two overall assessments was presented in box plots.

To determine the impact of potential confounding factors on the overall results, we also performed separate descriptive analyses according to profession, practical experience with AGREE I or II (number of guidelines appraised, experience in years), and any prior involvement in guideline development.

Before conducting the survey, we had formed the following three categories to assess the strength of the influence of the items on the two overall assessments and to enable clearer interpretation of the results: weak, medium, and strong influence (0–1, 2–3, and 4–5 points; median values).

## Results

### Response to online survey

**Fig. 1:**
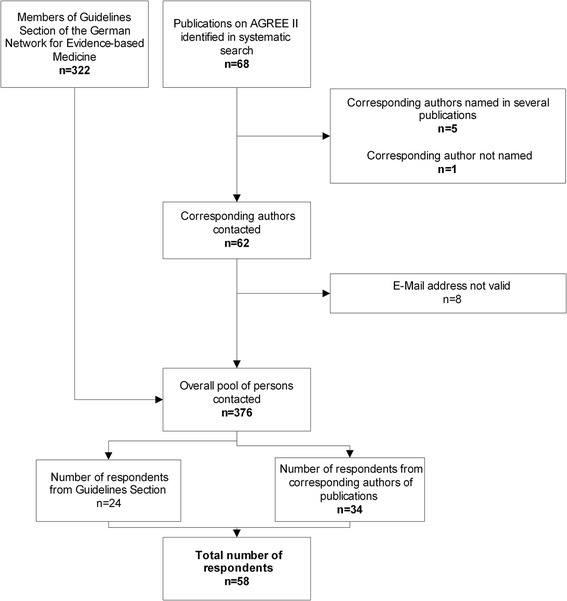
Flow chart of survey respondents

A total of 376 guideline users with valid e-mail addresses were contacted: the German version of the survey was sent to 322 members of DNEbM and the English version was sent to 54 corresponding authors of publications on guideline appraisals (Fig. 1). Fifty-eight of the 376 persons contacted (15.4%) participated in the survey (see the raw data in Additional file 2): 34 of the 54 corresponding authors of publications (63.0%) and 24 of the 322 DNEbM members (7.5%).

### Characteristics of respondents

Thirty-two (55.2%) of the 58 respondents were physicians of whom 10 (17.2%) were also methodological experts (Table 2). A further 10 respondents (17.2%) were solely methodological experts and 16 (27.6%) were from other professions (e.g. health scientists, pharmacologists, psychologists). 49 (84.5%) had previously performed guideline appraisals with AGREE I or II: 27 (46.6%) had performed less than 10 appraisals, nine (15.5%) had performed 10 to 20 appraisals and 13 (22.4%) had performed more than 20 appraisals.

**Table 2:** Characteristics of respondents

Characteristics	Respondents *N* = 58 (%)
Profession	
Physician	22 (37.9)
Physician/methodological expert	10 (17.2)
Methodological expert	10 (17.2)
Other	16 (27.6)
Knowledge of the AGREE II instrument	
Yes	51 (87.9)
No	7 (12.1)
Performance of appraisals using the AGREE I or II instrument	
Yes	49 (84.5)
No	9 (15.5)
Number of appraised guidelines using the AGREE I or II instrument^a^	
< 10 guidelines	27 (46.6)
10–20 guidelines	9 (15.5)
> 20 guidelines	13 (22.4)
Experience in years^a^	
< 1 year	6 (10.3)
1–5 years	35 (60.3)
> 5 years	8 (13.8)
Involvement in guideline development	
Yes	35 (60.3)
No	23 (39.7)
Purpose of conducting appraisals using the AGREE I or II instrument^b^	
Assessment of guideline quality	24 (41.4)
Development of guidelines	7 (12.1)
Writing of guideline synopses	7 (12.1)
Research	3 (5.2)
Adaptation of guidelines	2 (3.4)
Application in clinical practice	2 (3.4)
Further training	2 (3.4)
Development of knowledge tools	1 (1.7)
Publication of scientific articles	1 (1.7)
Project work	1 (1.7)
Updating of guidelines	1 (1.7)
No response	5 (8.6)

^a^Nine survey respondents (15.5%) did not answer this question

^b^The question was formulated as an open question; we summarized the response options presented here from the individual responses given. Some of the respondents provided more than one response

Six (10.3%) of the respondents had less than one year experience with AGREE I or II appraisals, 35 (60.3%) had one to five years’ experience, and eight (13.8%) had more than five years’ experience. 35 (60.3%) had already been involved in guideline development. The most commonly reported reason for application of AGREE I or II was appraisal of guideline quality (24 respondents, 41.4%) followed by development of guidelines (seven respondents; 12.1%) and writing of guideline synopses (seven; 12.1%).

### Open question on use of items and domains

Twenty-one of the 58 respondents (36.2%) answered the open question on which items they use for the overall assessment of guideline quality: 10 (17.2%) stated that all items were used in equal measure and one (1.7%) stated that no item was used. Nine respondents (15.5%) named domains, not items. All nine named Domain 3 (rigour of development); four named this domain as the only domain and five named Domain 3 in combination with other domains. The second most named domain was Domain 6 (editorial independence). Only one respondent (1.7%) specified items (Items 9 and 12 of Domain 3).

It should be noted that seven respondents reported that they had no knowledge of AGREE II. However, two of them still answered the further questions; it is unclear whether their first answer was incorrect or whether they provided answers without having knowledge of AGREE II. For this reason, both of these respondents were excluded from further analysis; the following results were thus provided by 51 respondents.

### Evaluation of the influence of the AGREE II items

Not all of the 51 respondents included in the analysis evaluated all items with regard to their influence on the two overall assessments of AGREE II: four respondents provided no such evaluation and two respondents discontinued their evaluation at Item 7 and Item 18.

**Fig. 2:**
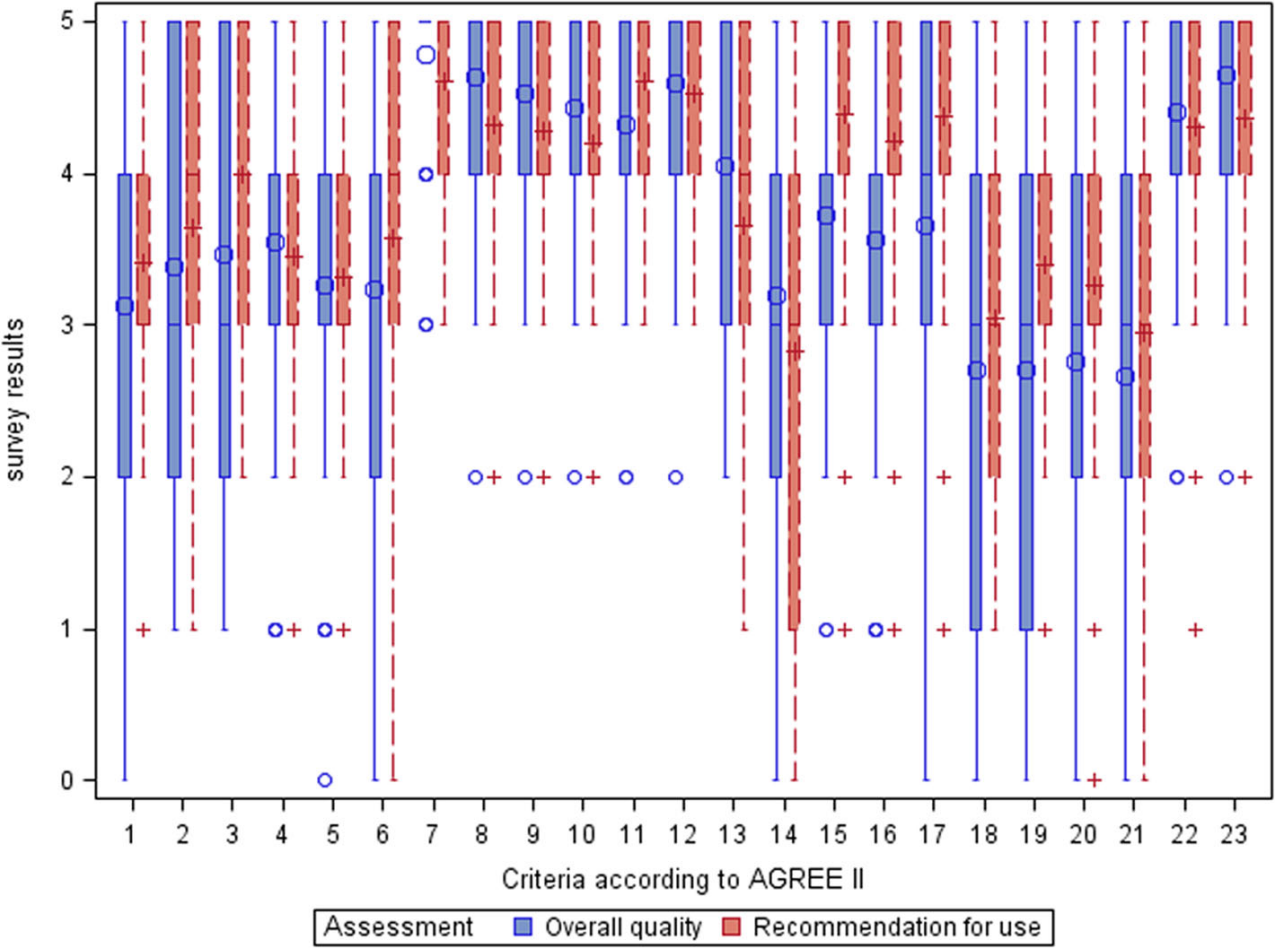
Influence of the AGREE II items on guideline quality and recommendation for use (overall data)

The boxplot shows great variations in the results for Items 1 to 3, 6, 14, 18, and 21 regarding both overall assessments (Fig. 2). For Items 19 und 20, the values vary greatly regarding guideline quality, but not regarding the recommendation for guideline use. The items with the strongest influence on the two overall assessments were reported to be Items 7 to 12 of Domain 3 (rigour of development) as well as both items (22 and 23) of Domain 6 (editorial independence). For Items 1, 15, 16 and 17–20, greater variations were notable for the influence on overall guideline quality than for the recommendation for use. A strong influence of these items can only be inferred for Items 15 to 17 of Domain 4 (clarity of presentation) with regard to the recommendation for use. The lowest scores were shown for the items of Domain 5 (applicability) and Item 14 of Domain 3, albeit with great variations.

The separate analyses of subgroups showed that the number of responses per subgroup (in most cases clearly fewer than 20 respondents) was too small to be able to draw valid conclusions on subgroup effects (data not shown). All in all, however, no marked deviations from the overall results were shown.

## Discussion

On the basis of a survey of guideline users, the aim of our analysis was to investigate how strongly the individual AGREE II items influenced the two overall assessments (overall guideline quality and recommendation for use). Our findings indicate that Items 7 to 12 (Domain 3; rigour of development) and both items of Domain 6 (editorial independence) had the strongest influence on the two overall assessments. In addition, Items 15 to 17 (clarity of presentation) had a strong influence on the recommendation for use. Great variations in respondents’ judgements were shown for the other items.

The importance of rigour of development (Domain 3) to guideline appraisers is not surprising, as this domain is regarded to be the strongest indicator of quality [10, 27], a high score for this domain indicating minimum bias and evidence-based guideline development [27]. The importance of editorial independence (Domain 6) highlights the relevance of conflicts of interest (COI) of guideline authors as a potential source of bias. Although the IOM clearly states that “To be *trustworthy*, guidelines should …[b]e based on an explicit and transparent process that minimizes distortions, biases, and conflicts of interest” [2], most guidelines fail to disclose authors’ COI, or if they do, numerous COI are reported [28–30].

In contrast to our systematic review [25], a strong influence of Domain 6, not Domain 5, was determined in the present analysis. This difference may have been caused by the different methods of data collection and data analysis: the data in our systematic review were based on actual applications of the AGREE II instrument whereas the data in the present analysis were based on more subjective assessments related to AGREE II collected by means of a survey. Therefore, some deviations in results are to be expected. We suggest considering Domain 6 in the weighting of results in order to achieve a more objective AGREE II assessment (see “Limitations”).

The finding that clarity of presentation (Domain 4) in a guideline had a strong influence on the recommendation for use is also not surprising, as “the main advantage of a well-reported guideline is that flaws in the methodology are more easily detected, so that inherent biases can be considered more explicitly and scrutinized by the potential users” [31].

### Previous and potential future approaches to overall assessments in AGREE II

The results of our survey show that the overall assessments of AGREE II are highly subjective and a standardized approach to reaching these assessments is lacking. This is in line with previous research: the publications identified in our systematic literature search showed considerable variations in how the results from appraisals with AGREE II are used to reach the two overall assessments. For instance, in contrast to the recommendation in AGREE II, some users apply cut-offs to distinguish between high and low-quality guidelines [20, 21, 27, 32–55]. Others calculate a score for overall quality from the six domain scores; however, this no longer represents a separate assessment as foreseen by AGREE II [24, 44, 49, 56–59]. Further users weight items or domains without clearly presenting how this weighting affects the overall assessments [33, 34, 37, 44, 45, 60–62]. This issue was also addressed by Alonso-Coello et al. in 2010 in their review on guideline quality, who noted that “… the validity of the overall assessment may be limited, as there were no clear rules on how to weigh the different domain scores in making a decision about whether or not to recommend the guidelines” [10]. As stated, it has not yet been investigated in detail to what extent the individual AGREE II items influence the two overall assessments; our recently published systematic review [25] and the present analysis thus represent the first research to investigate this question.

The AGREE II user manual does not require transparent reporting with regard to how users reach their overall assessments and the approach applied is thus at the discretion of the users. This means that it is unclear how and to what extent these assessments are influenced by the individual assessments of items and domains. To ensure a transparent approach, the AGREE II user manual could include an a-priori weighting of those items and domains that should have the strongest influence on the two overall assessments. This would mean specifying which items are more (or less) useful regarding the operationalization of the conduct of the two overall assessments. This weighting approach could be included in an update of AGREE II to achieve more transparent operationalization, thus increasing objectiveness and leading to more comparable results of different appraisals of the same guideline. Ultimately, this would help to distinguish more clearly between high and low-quality guidelines. Additionally, the weighting approach could be used in the development of a rapid appraisal instrument including only the most useful items for the two overall assessments, and thus help to save resources.

In this context one could consider the findings by Fervers et al. [31], who examined characteristics of guidelines and guideline developing organizations to identify predictors of high-quality guidelines. They identified the availability of background information, that is, “explicit and detailed information about the objectives and context of the guideline development, including the methods used, and the people and organizations involved in the development process” [31] as the strongest predictor of guideline quality, in particular for Domain 3 (rigour of development). The components cited could be used to help weight items in AGREE II.

### Limitations

Our analysis is the first to investigate the influence of individual AGREE II items on overall guideline quality and recommendation for use. However, due to the low response rate of the survey (15.5%), only indications but no robust conclusions can be drawn from our findings. We had contacted members of the guideline section of a German scientific network, as we had expected a high response rate from this large pool of guideline users. However, the opposite was the case; the response rate in this group was actually far lower than in the group of authors of guideline appraisal articles (7.5% vs. 63.0%). One potential explanation could be that not all members of the guideline section of the German scientific network are actually involved in guideline development, but belong to this section due to their basic interest in clinical practice guidelines. Furthermore, some members of this section also belong to other working groups, so it is possible that some responses represent feedback from a whole working group rather than from a single respondent. In addition, non-responses are not necessarily limited to individual respondents, but can be associated with whole organizations choosing not to participate in a study [63].

In addition, German guideline appraisers primarily use the German adaptation of AGREE I (DELBI, [64]) and not the English-language instrument AGREE II – we did not consider DELBI in our survey, as it is not validated and is based on AGREE I. In contrast, the guideline appraisal articles identified in our systematic search referred primarily to AGREE II and one can thus assume a greater interest of these respondents in the survey. A further reason for the overall low response rate could be the type of survey conducted; web-based surveys often have lower response rates than those conducted by letter or phone [65].

Although nearly two-thirds of the respondents were not methodological experts, the results show a strong influence of Domain 3 (rigour of development); in our opinion a higher response rate including a higher proportion of methodological experts would therefore not necessarily have changed the results of the survey. However, we did not systematically assess the non-responses and our comments above are thus based on assumptions: ultimately, the extent to which the responses of the non-respondents would have changed the initial results is unclear and we cannot exclude potential bias.

## Conclusions

The results of our survey indicate that in guideline appraisals using AGREE II, items representing the rigour of guideline development and the editorial independence of authors seem to have the strongest influence on the overall assessment of guideline quality and recommendation for use. In addition, items representing the clarity of presentation have a strong influence on the recommendation for use. Great variations in respondents’ judgements exist regarding the other AGREE II items.

In order to ensure a transparent and consistent approach to reaching the two overall assessments, besides encouraging transparent reporting, we suggest the inclusion of a recommendation in the AGREE II user manual on how to consider item and domain scores. For instance, the user manual could include an a-priori weighting of those items and domains that should have the strongest influence on the 2 overall assessments so as to help distinguish more clearly between high and low-quality guidelines.

In addition, the weighting approach could be used in the development of a short (and economical) form of guideline appraisal including only the most important items and domains. In the next update of AGREE II, our study could thus help to contribute to determining which items and domains are most important for the operationalization of the two overall assessments. The relevance of the two overall assessments within AGREE II could thereby be further specified.

## Additional files

Additional file 1: Questionnaire. (PDF 42 kb)

Additional file 2: Results (raw data of 58 respondents) of the assessment of the strength of the potential influence of the AGREE II items on the two overall assessments. (PDF 308 kb)

## Abbreviations

AGREE: Appraisal of Guidelines for Research and Evaluation
COI: Conflict of interest
DELBI: German Guideline Appraisal Instrument
DNEbM: German Network for Evidence-based Medicine
IFOM: Institute for Research in Operative Medicine
IOM: Institute of Medicine
IQWiG: Institute for Quality and Efficiency in Healthcare
MDS: Medical Advisory Service of the German Social Health Insurance
PASW: Predictive Analysis SoftWare
SPSS: Superior Performing Software Systems
