# Supplementary material for: Guideline appraisal with AGREE II: online survey of the potential influence of AGREE II items on overall assessment of guideline quality and recommendation for use
Source: BMC Health Serv Res. 2018 Feb 27;18:143. doi: 10.1186/s12913-018-2954-8 (PMC5828401; doi:10.1186/s12913-018-2954-8)
Supplement: Supplementary file 1 — Questionnaire. (PDF 42 kb) [file 12913_2018_2954_MOESM1_ESM.pdf]

1 Background of the survey

As a PhD student at the University of Witten/Herdecke and a Research Associate at the German Institute for Quality and Efficiency in Health Care (IQWiG), I am currently working on the topic “Guideline appraisal according to AGREE II: Systematic review of the current evidence base on the handling of the overall assessment.” The PhD project also comprises an empirical investigation of the relationship between the individual assessments and the two overall assessments included in the AGREE II instrument.

For this purpose, in an online survey I would like to investigate how strongly the individual criteria (“items”) of the AGREE II instrument are related to the two overall assessments.

As you are a guideline expert/appraiser and the author of scientific publications on methodological guideline appraisal using AGREE II, I kindly ask you to answer the questions listed below.

With your participation you would provide a valuable contribution to the success of the project and would also potentially contribute to the incorporation of new scientific findings in the appraisal of guideline quality.

The publication of this PhD project will include the list of survey participants. However, the results of the survey will be anonymized, i.e. there will be no allocation of participants or their institutions to the results of the survey.

My aim is to obtain the most comprehensive information base possible for the project. I would thus be very grateful if you could respond to the questions and send the completed questionnaire back to me by XX.XX.201X.

Thank you very much for your support

Yours sincerely

Wiebke Hoffmann-Eßer

Explanation of the assessment scale

The question: “How strongly do the individual items of the AGREE instrument influence 1) the overall methodological assessment according to AGREE II and 2) the recommendation to use the guideline?” is to be answered by ticking boxes on a scale of 0 (= no influence) to 5 (= very strong influence).

The scale of 0 to 5 indicates how weak (0= no influence) or how strong (5= very strong influence) the influence of the respective individual AGREE item is on the overall assessment(s) according to AGREE II.

2 Questionnaire

Only summarized, i.e. no individual, data will be published. It is thus not possible to identify individual assessors.

2.1 Knowledge of the AGREE instrument

I know the AGREE II instrument for appraisal of guideline quality.

Yes ☐ No ☐

I myself have already performed appraisals using the AGREE instrument.

Yes ☐ No ☐

If yes: how many? 0-10 / 10-20 / more than 20?

< 10 ☐ 10-20 ☐ > 20 ☐

If yes: For how many years have you been conducting appraisals using the AGREE instrument?

< 1 ☐ 1-5 ☐ > 5 ☐

If yes: For which purpose did you conduct appraisals using the AGREE instrument?

____
____
____

Have you also been involved in the development of a guideline?

Yes ☐ No ☐

What is your profession?

Physician ☐ Methodological expert ☐ Other ☐

If you ticked “Other”, could you please specify?

____

____

2.2 Assessment of items

“The AGREE II consists of 23 key items organized within 6 domains followed by 2 global rating items (“Overall Assessment”). Each domain captures a unique dimension of guideline quality.…Upon completing the 23 items, AGREE II users will provide 2 overall assessments of the guideline. The overall assessment requires the user to make a judgment as to the quality of the guideline, taking into account the criteria considered in the assessment process. The user is also asked whether he/she would recommend use of the guideline” [AGREE Next Steps Consortium. Appraisal of guidelines for research & evaluation II: AGREE II instrument [online]. 05.2009

How strongly do the individual items of the AGREE instrument influence 1) the methodological overall assessment according to AGREE II and 2) the recommendation to use the guideline? For each item, please tick the respective box on the scale. This scale ranges from 0 (= no influence) to 5 (= very strong influence).

Which items do you use for the overall appraisal of the methodological quality of guidelines?

____

____

____

Domain 1: Scope and Purpose

AGREE Item 1: The overall objective(s) of the guideline is (are) specifically described.

Relevance of Item 1 for the **overall methodological assessment**?

No influence					very strong
**0**	**1**	**2**	**3**	**4**	**5**
☐	☐	☐	☐	☐	☐

Relevance of Item 1 for the **recommendation to use the guideline**?

No influence					very strong
**0**	**1**	**2**	**3**	**4**	**5**
☐	☐	☐	☐	☐	☐

AGREE Item 2: The health question(s) covered by the guideline (is (are) specifically described.

Relevance of Item 2 for the **overall methodological assessment**?

No influence					very strong
0	1	2	3	4	5
☐	☐	☐	☐	☐	☐

Relevance of Item 2 for the recommendation **to use the guideline**?

No influence					very strong
0	1	2	3	4	5
☐	☐	☐	☐	☐	☐

AGREE Item 3: The population (patients, public, etc.) to whom the guideline is meant to apply is specifically described.

Relevance of Item 3 for the **overall methodological assessment**?

No influence					very strong
0	1	2	3	4	5
☐	☐	☐	☐	☐	☐

Relevance of Item 3 for the recommendation **to use the guideline**?

No influence					very strong
0	1	2	3	4	5
☐	☐	☐	☐	☐	☐

<u>Domain 2: Stakeholder Involvement</u>

AGREE Item 4: The guideline development group includes individuals from all relevant professional groups.

Relevance of Item 4 for the **overall methodological assessment**?

No influence					very strong
0	1	2	3	4	5
☐	☐	☐	☐	☐	☐

Relevance of Item 4 for the recommendation **to use the guideline**?

No influence					very strong
0	1	2	3	4	5
☐	☐	☐	☐	☐	☐

AGREE Item 5: The views and preferences of the target population (patients, public etc.) have been sought.

Relevance of Item 5 for the **overall methodological assessment**?

No influence					very strong
0	1	2	3	4	5
☐	☐	☐	☐	☐	☐

Relevance of Item 5 for the recommendation **to use the guideline**?

No influence					very strong
0	1	2	3	4	5
☐	☐	☐	☐	☐	☐

AGREE Item 6: The target users of the guideline are clearly defined.

Relevance of Item 6 for the **overall methodological assessment**?

No influence					very strong
0	1	2	3	4	5
☐	☐	☐	☐	☐	☐

Relevance of Item 6 for the recommendation **to use the guideline**?

No influence					very strong
0	1	2	3	4	5
☐	☐	☐	☐	☐	☐

<u>Domain 3: Rigour of Development</u>

AGREE Item 7: Systematic methods were used to search for evidence.

Relevance of Item 7 for the **overall methodological assessment**?

No influence					very strong
0	1	2	3	4	5
☐	☐	☐	☐	☐	☐

Relevance of Item 7 for the recommendation **to use the guideline**?

No influence					very strong
0	1	2	3	4	5

☐ ☐ ☐ ☐ ☐ ☐

AGREE Item 8: The criteria for selecting the evidence are clearly described.

Relevance of Item 8 for the **overall methodological assessment**?

No influence **very strong**

**0**	**1**	**2**	**3**	**4**	**5**
☐	☐	☐	☐	☐	☐

Relevance of Item 8 for the recommendation **to use the guideline**?

No influence **very strong**

**0**	**1**	**2**	**3**	**4**	**5**
☐	☐	☐	☐	☐	☐

AGREE Item 9: The strengths and limitations of the body of evidence are clearly described.

Relevance of Item 9 for the **overall methodological assessment**?

No influence **very strong**

**0**	**1**	**2**	**3**	**4**	**5**
☐	☐	☐	☐	☐	☐

Relevance of Item 9 for the recommendation **to use the guideline**?

No influence **very strong**

**0**	**1**	**2**	**3**	**4**	**5**
☐	☐	☐	☐	☐	☐

AGREE Item 10: The methods for formulating the recommendations are clearly described.

Relevance of Item 10 for the **overall methodological assessment**?

No influence **very strong**

**0**	**1**	**2**	**3**	**4**	**5**
☐	☐	☐	☐	☐	☐

Relevance of Item 10 for the recommendation **to use the guideline**?

No influence **very strong**

**0**	**1**	**2**	**3**	**4**	**5**
☐	☐	☐	☐	☐	☐

AGREE Item 11: The health benefits, side effects, and risks have been considered in formulating the recommendations.

Relevance of Item 11 for the **overall methodological assessment**?

**No influence**					**very strong**
**0**	**1**	**2**	**3**	**4**	**5**
☐	☐	☐	☐	☐	☐

Relevance of Item 11 for the recommendation **to use the guideline**?

**No influence**					**very strong**
**0**	**1**	**2**	**3**	**4**	**5**
☐	☐	☐	☐	☐	☐

AGREE Item 12: There is an explicit link between the recommendations and the supporting evidence.

Relevance of Item 12 for the **overall methodological assessment**?

**No influence**					**very strong**
**0**	**1**	**2**	**3**	**4**	**5**
☐	☐	☐	☐	☐	☐

Relevance of Item 12 for the recommendation **to use the guideline**?

**No influence**					**very strong**
**0**	**1**	**2**	**3**	**4**	**5**
☐	☐	☐	☐	☐	☐

AGREE Item 13: The guideline has been externally reviewed by experts prior to its publication.

Relevance of Item 13 for the **overall methodological assessment**?

**No influence**					**very strong**
**0**	**1**	**2**	**3**	**4**	**5**
☐	☐	☐	☐	☐	☐

Relevance of Item 13 for the recommendation **to use the guideline**?

**No influence**					**very strong**
**0**	**1**	**2**	**3**	**4**	**5**
☐	☐	☐	☐	☐	☐

AGREE Item 14: A procedure for updating the guidelines is provided.

Relevance of Item 14 for the **overall methodological assessment**?

No influence					very strong
0	1	2	3	4	5
☐	☐	☐	☐	☐	☐

Relevance of Item 14 for the recommendation **to use the guideline**?

No influence					very strong
0	1	2	3	4	5
☐	☐	☐	☐	☐	☐

Domain 4: Clarity of Presentation

AGREE Item 15: The recommendations are specific and unambiguous.

Relevance of Item 15 for the **overall methodological assessment**?

No influence					very strong
0	1	2	3	4	5
☐	☐	☐	☐	☐	☐

Relevance of Item 15 for the recommendation **to use the guideline**?

No influence					very strong
0	1	2	3	4	5
☐	☐	☐	☐	☐	☐

AGREE Item 16: The different options for management of the condition or health issue are clearly presented.

Relevance of Item 16 for the **overall methodological assessment**?

No influence					very strong
0	1	2	3	4	5
☐	☐	☐	☐	☐	☐

Relevance of Item 16 for the recommendation **to use the guideline**?

No influence					very strong
0	1	2	3	4	5
☐	☐	☐	☐	☐	☐

AGREE Item 17: Key recommendations are easily identifiable.

Relevance of Item 17 for the **overall methodological assessment**?

No influence				very strong	
**0**	**1**	**2**	**3**	**4**	**5**
☐	☐	☐	☐	☐	☐

Relevance of Item 17 for the recommendation **to use the guideline**?

No influence				very strong	
**0**	**1**	**2**	**3**	**4**	**5**
☐	☐	☐	☐	☐	☐

Domain 5: Applicability

AGREE Item 18: The guideline describes facilitators and barriers to its application.

Relevance of Item 18 for the **overall methodological assessment**?

No influence				very strong	
**0**	**1**	**2**	**3**	**4**	**5**
☐	☐	☐	☐	☐	☐

Relevance of Item 18 for the recommendation **to use the guideline**?

No influence				very strong	
**0**	**1**	**2**	**3**	**4**	**5**
☐	☐	☐	☐	☐	☐

AGREE Item 19: The guideline provides advice and/or tools on how the recommendations can be put into practice.

Relevance of Item 19 for the **overall methodological assessment**?

No influence				very strong	
**0**	**1**	**2**	**3**	**4**	**5**
☐	☐	☐	☐	☐	☐

Relevance of Item 19 for the recommendation **to use the guideline**?

No influence				very strong	
**0**	**1**	**2**	**3**	**4**	**5**
☐	☐	☐	☐	☐	☐

AGREE Item 20: The potential resource implications of applying the recommendations have been considered.

Relevance of Item 20 for the **overall methodological assessment**?

No influence					very strong
0	1	2	3	4	5
☐	☐	☐	☐	☐	☐

Relevance of Item 20 for the recommendation **to use the guideline**?

No influence					very strong
0	1	2	3	4	5
☐	☐	☐	☐	☐	☐

AGREE Item 21: The guideline presents monitoring and/or auditing criteria.

Relevance of Item 21 for the **overall methodological assessment**?

No influence					very strong
0	1	2	3	4	5
☐	☐	☐	☐	☐	☐

Relevance of Item 21 for the recommendation **to use the guideline**?

No influence					very strong
0	1	2	3	4	5
☐	☐	☐	☐	☐	☐

Domain 6: Editorial Independence

AGREE Item 22: The views of the funding body have not influenced the content of the guideline.

Relevance of Item 22 for the **overall methodological assessment**?

No influence					very strong
0	1	2	3	4	5
☐	☐	☐	☐	☐	☐

Relevance of Item 22 for the recommendation **to use the guideline**?

No influence					very strong
0	1	2	3	4	5
☐	☐	☐	☐	☐	☐

AGREE Item 23: Competing interests of guideline development group members have been recorded and addressed.

Relevance of Item 23 for the **overall methodological assessment**?

No influence					very strong
0	1	2	3	4	5
☐	☐	☐	☐	☐	☐

Relevance of Item 23 for the recommendation **to use the guideline**?

No influence					very strong
0	1	2	3	4	5
☐	☐	☐	☐	☐	☐

Further comments on the assessments above:

2.3 If you would like to be informed about the results of the project, please send an e-mail to

wiebke.hoffmann-esser@iqwig.de
