# Supplementary material for: Guideline appraisal with AGREE II: online survey of the potential influence of AGREE II items on overall assessment of guideline quality and recommendation for use
Source: BMC Health Serv Res. 2018 Feb 27;18:143. doi: 10.1186/s12913-018-2954-8 (PMC5828401; doi:10.1186/s12913-018-2954-8)
Supplement: Supplementary file 2 — Results (raw data of 58 respondents) of the assessment of the strength of the potential influence of the AGREE II items on the two overall assessments. (PDF 308 kb) [file 12913_2018_2954_MOESM2_ESM.pdf]

## Results (raw data of 58 respondents) of the assessment of the strength of the potential influence of the AGREE II items on the two overall assessments

| No of respondent | Profession | Knowledge of the AGREE II instrument | Performance of appraisals using the AGREE I or II instrument | Number of appraised guidelines using the AGREE I or II instrument | Experience in years | Involvement in guideline development | Purpose of conducting appraisals using the AGREE I or II instrument | AGREE II Items | Assessment of the influence on methodological quality | Assessment of the influence on recommendation for use |
|---|---|---|---|---|---|---|---|---|---|---|
| **1** | methodological expert | yes | 10-20 guidelines | - | - | yes | - | **1** | 0 | 2 |
| | | | | | | | | **2** | 4 | 1 |
| | | | | | | | | **3** | 5 | 5 |
| | | | | | | | | **4** | 5 | 5 |
| | | | | | | | | **5** | 5 | 5 |
| | | | | | | | | **6** | 0 | 5 |
| | | | | | | | | **7** | 5 | 5 |
| | | | | | | | | **8** | 5 | 5 |
| | | | | | | | | **9** | 5 | 5 |
| | | | | | | | | **10** | 5 | 5 |
| | | | | | | | | **11** | 5 | 5 |
| | | | | | | | | **12** | 5 | 3 |
| | | | | | | | | **13** | 5 | 5 |
| | | | | | | | | **14** | 5 | 5 |
| | | | | | | | | **15** | 3 | 5 |
| | | | | | | | | **16** | 5 | 5 |
| | | | | | | | | **17** | 5 | 5 |
| | | | | | | | | **18** | 0 | 5 |
| | | | | | | | | **19** | 0 | 5 |

| No of respondent | Profession | Knowledge of the AGREE II instrument | Performance of appraisals using the AGREE I or II instrument | Number of appraised guidelines using the AGREE I or II instrument | Experience in years | Involvement in guideline development | Purpose of conducting appraisals using the AGREE I or II instrument | AGREE II Items | Assessment of the influence on methodological quality | Assessment of the influence on recommendation for use |
|---|---|---|---|---|---|---|---|---|---|---|
| | | | | | | | | **20** | 0 | 5 |
| | | | | | | | | **21** | 0 | 5 |
| | | | | | | | | **22** | 5 | 5 |
| | | | | | | | | **23** | 5 | 5 |
| **2** | Physician | yes | yes | < 10 guidelines | 1-5 years | yes | Assessment of guideline quality | **1** | 3 | 3 |
| | | | | | | | | **2** | 3 | 3 |
| | | | | | | | | **3** | 3 | 3 |
| | | | | | | | | **4** | 3 | 3 |
| | | | | | | | | **5** | 3 | 3 |
| | | | | | | | | **6** | 3 | 3 |
| | | | | | | | | **7** | 3 | 3 |
| | | | | | | | | **8** | 3 | 3 |
| | | | | | | | | **9** | 3 | 3 |
| | | | | | | | | **10** | 3 | 3 |
| | | | | | | | | **11** | 3 | 3 |
| | | | | | | | | **12** | 3 | 3 |
| | | | | | | | | **13** | 3 | 3 |
| | | | | | | | | **14** | 3 | 3 |
| | | | | | | | | **15** | 3 | 3 |
| | | | | | | | | **16** | 3 | 3 |
| | | | | | | | | **17** | 3 | 3 |

| No of respondent | Profession | Knowledge of the AGREE II instrument | Performance of appraisals using the AGREE I or II instrument | Number of appraised guidelines using the AGREE I or II instrument | Experience in years | Involvement in guideline development | Purpose of conducting appraisals using the AGREE I or II instrument | AGREE II Items | Assessment of the influence on methodological quality | Assessment of the influence on recommendation for use |
|---|---|---|---|---|---|---|---|---|---|---|
| | | | | | | | | **18** | 3 | 3 |
| | | | | | | | | **19** | 3 | 3 |
| | | | | | | | | **20** | 3 | 3 |
| | | | | | | | | **21** | 3 | 3 |
| | | | | | | | | **22** | 3 | 3 |
| | | | | | | | | **23** | 3 | 3 |
| **3**[b] | Physician | no | no | - | - | no | - | **1** | - | - |
| | | | | | | | | **2** | - | - |
| | | | | | | | | **3** | - | - |
| | | | | | | | | **4** | - | - |
| | | | | | | | | **5** | - | - |
| | | | | | | | | **6** | - | - |
| | | | | | | | | **7** | - | - |
| | | | | | | | | **8** | - | - |
| | | | | | | | | **9** | - | - |
| | | | | | | | | **10** | - | - |
| | | | | | | | | **11** | - | - |
| | | | | | | | | **12** | - | - |
| | | | | | | | | **13** | - | - |
| | | | | | | | | **14** | - | - |
| | | | | | | | | **15** | - | - |

| No of respondent | Profession | Knowledge of the AGREE II instrument | Performance of appraisals using the AGREE I or II instrument | Number of appraised guidelines using the AGREE I or II instrument | Experience in years | Involvement in guideline development | Purpose of conducting appraisals using the AGREE I or II instrument | AGREE II Items | Assessment of the influence on methodological quality | Assessment of the influence on recommendation for use |
|---|---|---|---|---|---|---|---|---|---|---|
| | | | | | | | | **16** | - | - |
| | | | | | | | | **17** | - | - |
| | | | | | | | | **18** | - | - |
| | | | | | | | | **19** | - | - |
| | | | | | | | | **20** | - | - |
| | | | | | | | | **21** | - | - |
| | | | | | | | | **22** | - | - |
| | | | | | | | | **23** | - | - |
| **4** | methodological expert | yes | yes | 10-20 guidelines | 1-5 years | yes | Assessment of guideline, Writing of guideline synopses | **1** | 0 | 3 |
| | | | | | | | | **2** | 1 | 3 |
| | | | | | | | | **3** | 1 | 4 |
| | | | | | | | | **4** | 1 | 4 |
| | | | | | | | | **5** | 0 | 2 |
| | | | | | | | | **6** | 0 | 3 |
| | | | | | | | | **7** | 5 | 5 |
| | | | | | | | | **8** | 5 | 5 |
| | | | | | | | | **9** | 5 | 4 |
| | | | | | | | | **10** | 4 | 4 |
| | | | | | | | | **11** | 3 | 4 |
| | | | | | | | | **12** | 4 | 3 |
| | | | | | | | | **13** | 3 | 1 |

| No of respondent | Profession | Knowledge of the AGREE II instrument | Performance of appraisals using the AGREE I or II instrument | Number of appraised guidelines using the AGREE I or II instrument | Experience in years | Involvement in guideline development | Purpose of conducting appraisals using the AGREE I or II instrument | AGREE II Items | Assessment of the influence on <u>methodological quality</u> | Assessment of the influence on <u>recommendation for use</u> |
|---|---|---|---|---|---|---|---|---|---|---|
| | | | | | | | | **14** | 1 | 0 |
| | | | | | | | | **15** | 3 | 5 |
| | | | | | | | | **16** | 1 | 3 |
| | | | | | | | | **17** | 1 | 4 |
| | | | | | | | | **18** | 0 | 2 |
| | | | | | | | | **19** | 0 | 4 |
| | | | | | | | | **20** | 0 | 3 |
| | | | | | | | | **21** | 0 | 3 |
| | | | | | | | | **22** | 3 | 4 |
| | | | | | | | | **23** | 3 | 4 |
| **5**[a] | Physician | yes | yes | < 10 guidelines | 1-5 years | yes | Development of guidelines | **1** | - | - |
| | | | | | | | | **2** | - | - |
| | | | | | | | | **3** | - | - |
| | | | | | | | | **4** | - | - |
| | | | | | | | | **5** | - | - |
| | | | | | | | | **6** | - | - |
| | | | | | | | | **7** | - | - |
| | | | | | | | | **8** | - | - |
| | | | | | | | | **9** | - | - |
| | | | | | | | | **10** | - | - |
| | | | | | | | | **11** | - | - |

| No of respondent | Profession | Knowledge of the AGREE II instrument | Performance of appraisals using the AGREE I or II instrument | Number of appraised guidelines using the AGREE I or II instrument | Experience in years | Involvement in guideline development | Purpose of conducting appraisals using the AGREE I or II instrument | AGREE II Items | Assessment of the influence on methodological quality | Assessment of the influence on recommendation for use |
|---|---|---|---|---|---|---|---|---|---|---|
| | | | | | | | | **12** | - | - |
| | | | | | | | | **13** | - | - |
| | | | | | | | | **14** | - | - |
| | | | | | | | | **15** | - | - |
| | | | | | | | | **16** | - | - |
| | | | | | | | | **17** | - | - |
| | | | | | | | | **18** | - | - |
| | | | | | | | | **19** | - | - |
| | | | | | | | | **20** | - | - |
| | | | | | | | | **21** | - | - |
| | | | | | | | | **22** | - | - |
| | | | | | | | | **23** | - | - |
| **6**[a] | Physician | yes | yes | < 10 guidelines | 1-5 years | yes | - | **1** | - | - |
| | | | | | | | | **2** | - | - |
| | | | | | | | | **3** | - | - |
| | | | | | | | | **4** | - | - |
| | | | | | | | | **5** | - | - |
| | | | | | | | | **6** | - | - |
| | | | | | | | | **7** | - | - |
| | | | | | | | | **8** | - | - |
| | | | | | | | | **9** | - | - |

| No of respondent | Profession | Knowledge of the AGREE II instrument | Performance of appraisals using the AGREE I or II instrument | Number of appraised guidelines using the AGREE I or II instrument | Experience in years | Involvement in guideline development | Purpose of conducting appraisals using the AGREE I or II instrument | AGREE II Items | Assessment of the influence on methodological quality | Assessment of the influence on recommendation for use |
|---|---|---|---|---|---|---|---|---|---|---|
| | | | | | | | | **10** | - | - |
| | | | | | | | | **11** | - | - |
| | | | | | | | | **12** | - | - |
| | | | | | | | | **13** | - | - |
| | | | | | | | | **14** | - | - |
| | | | | | | | | **15** | - | - |
| | | | | | | | | **16** | - | - |
| | | | | | | | | **17** | - | - |
| | | | | | | | | **18** | - | - |
| | | | | | | | | **19** | - | - |
| | | | | | | | | **20** | - | - |
| | | | | | | | | **21** | - | - |
| | | | | | | | | **22** | - | - |
| | | | | | | | | **23** | - | - |
| **7** | Physician / methodological expert | yes | yes | < 10 guidelines | 1-5 years | yes | Development of guidelines | **1** | 5 | 5 |
| | | | | | | | | **2** | 5 | 5 |
| | | | | | | | | **3** | 5 | 5 |
| | | | | | | | | **4** | 1 | 1 |
| | | | | | | | | **5** | 4 | 4 |
| | | | | | | | | **6** | 4 | 4 |
| | | | | | | | | **7** | 5 | 5 |

| No of respondent | Profession | Knowledge of the AGREE II instrument | Performance of appraisals using the AGREE I or II instrument | Number of appraised guidelines using the AGREE I or II instrument | Experience in years | Involvement in guideline development | Purpose of conducting appraisals using the AGREE I or II instrument | AGREE II Items | Assessment of the influence on methodological quality | Assessment of the influence on recommendation for use |
|---|---|---|---|---|---|---|---|---|---|---|
| | | | | | | | | **8** | 5 | 5 |
| | | | | | | | | **9** | 5 | 5 |
| | | | | | | | | **10** | 5 | 5 |
| | | | | | | | | **11** | 4 | 5 |
| | | | | | | | | **12** | 4 | 4 |
| | | | | | | | | **13** | 3 | 3 |
| | | | | | | | | **14** | 2 | 1 |
| | | | | | | | | **15** | 1 | 5 |
| | | | | | | | | **16** | 1 | 5 |
| | | | | | | | | **17** | 1 | 5 |
| | | | | | | | | **18** | 1 | 1 |
| | | | | | | | | **19** | 1 | 1 |
| | | | | | | | | **20** | 0 | 0 |
| | | | | | | | | **21** | 0 | 0 |
| | | | | | | | | **22** | 4 | 5 |
| | | | | | | | | **23** | 4 | 5 |
| **8**[b] | other | no | no | - | - | no | - | **1** | - | - |
| | | | | | | | | **2** | - | - |
| | | | | | | | | **3** | - | - |
| | | | | | | | | **4** | - | - |
| | | | | | | | | **5** | - | - |

| **No of respondent** | **Profession** | **Knowledge of the AGREE II instrument** | **Performance of appraisals using the AGREE I or II instrument** | **Number of appraised guidelines using the AGREE I or II instrument** | **Experience in years** | **Involvement in guideline development** | **Purpose of conducting appraisals using the AGREE I or II instrument** | **AGREE II Items** | **Assessment of the influence on <u>methodological quality</u>** | **Assessment of the influence on <u>recommendation for use</u>** |
|---|---|---|---|---|---|---|---|---|---|---|
| | | | | | | | | **6** | - | - |
| | | | | | | | | **7** | - | - |
| | | | | | | | | **8** | - | - |
| | | | | | | | | **9** | - | - |
| | | | | | | | | **10** | - | - |
| | | | | | | | | **11** | - | - |
| | | | | | | | | **12** | - | - |
| | | | | | | | | **13** | - | - |
| | | | | | | | | **14** | - | - |
| | | | | | | | | **15** | - | - |
| | | | | | | | | **16** | - | - |
| | | | | | | | | **17** | - | - |
| | | | | | | | | **18** | - | - |
| | | | | | | | | **19** | - | - |
| | | | | | | | | **20** | - | - |
| | | | | | | | | **21** | - | - |
| | | | | | | | | **22** | - | - |
| | | | | | | | | **23** | - | - |
| **9** | other | yes | yes | > 20 guidelines | 1-5 years | no | Project work | **1** | 2 | 3 |
| | | | | | | | | **2** | 3 | 3 |
| | | | | | | | | **3** | 2 | 2 |

| **No of respondent** | **Profession** | **Knowledge of the AGREE II instrument** | **Performance of appraisals using the AGREE I or II instrument** | **Number of appraised guidelines using the AGREE I or II instrument** | **Experience in years** | **Involvement in guideline development** | **Purpose of conducting appraisals using the AGREE I or II instrument** | **AGREE II Items** | **Assessment of the influence on <u>methodological quality</u>** | **Assessment of the influence on <u>recommendation for use</u>** |
|---|---|---|---|---|---|---|---|---|---|---|
| | | | | | | | | **4** | 4 | 3 |
| | | | | | | | | **5** | 4 | 3 |
| | | | | | | | | **6** | 3 | 3 |
| | | | | | | | | **7** | 4 | 4 |
| | | | | | | | | **8** | 4 | 3 |
| | | | | | | | | **9** | 4 | 3 |
| | | | | | | | | **10** | 4 | 4 |
| | | | | | | | | **11** | 4 | 4 |
| | | | | | | | | **12** | 4 | 4 |
| | | | | | | | | **13** | 3 | 3 |
| | | | | | | | | **14** | 4 | 3 |
| | | | | | | | | **15** | 3 | 5 |
| | | | | | | | | **16** | 3 | 5 |
| | | | | | | | | **17** | 3 | 5 |
| | | | | | | | | **18** | 3 | 2 |
| | | | | | | | | **19** | 3 | 2 |
| | | | | | | | | **20** | 2 | 2 |
| | | | | | | | | **21** | 4 | 3 |
| | | | | | | | | **22** | 4 | 4 |
| | | | | | | | | **23** | 5 | 4 |
| **10** | methodological | yes | yes | < 10 | 1-5 years | yes | - | **1** | 1 | 1 |

| **No of respondent** | **Profession** | **Knowledge of the AGREE II instrument** | **Performance of appraisals using the AGREE I or II instrument** | **Number of appraised guidelines using the AGREE I or II instrument** | **Experience in years** | **Involvement in guideline development** | **Purpose of conducting appraisals using the AGREE I or II instrument** | **AGREE II Items** | **Assessment of the influence on methodological quality** | **Assessment of the influence on recommendation for use** |
|---|---|---|---|---|---|---|---|---|---|---|
| | expert | | | guidelines | | | | **2** | 2 | 2 |
| | | | | | | | | **3** | 1 | 2 |
| | | | | | | | | **4** | 2 | 2 |
| | | | | | | | | **5** | 2 | 2 |
| | | | | | | | | **6** | 0 | 0 |
| | | | | | | | | **7** | 5 | 5 |
| | | | | | | | | **8** | 4 | 4 |
| | | | | | | | | **9** | 4 | 4 |
| | | | | | | | | **10** | 3 | 3 |
| | | | | | | | | **11** | 5 | 5 |
| | | | | | | | | **12** | 5 | 5 |
| | | | | | | | | **13** | 3 | 3 |
| | | | | | | | | **14** | 0 | 0 |
| | | | | | | | | **15** | 1 | 1 |
| | | | | | | | | **16** | 2 | 2 |
| | | | | | | | | **17** | 1 | 1 |
| | | | | | | | | **18** | 1 | 1 |
| | | | | | | | | **19** | 2 | 2 |
| | | | | | | | | **20** | 1 | 1 |
| | | | | | | | | **21** | 2 | 2 |
| | | | | | | | | **22** | 2 | 2 |

| No of respondent | Profession | Knowledge of the AGREE II instrument | Performance of appraisals using the AGREE I or II instrument | Number of appraised guidelines using the AGREE I or II instrument | Experience in years | Involvement in guideline development | Purpose of conducting appraisals using the AGREE I or II instrument | AGREE II Items | Assessment of the influence on methodological quality | Assessment of the influence on recommendation for use |
|---|---|---|---|---|---|---|---|---|---|---|
| | | | | | | | | 23 | 5 | 5 |
| **11[b]** | other | no | no | - | - | no | - | **1** | - | - |
| | | | | | | | | **2** | - | - |
| | | | | | | | | **3** | - | - |
| | | | | | | | | **4** | - | - |
| | | | | | | | | **5** | - | - |
| | | | | | | | | **6** | - | - |
| | | | | | | | | **7** | - | - |
| | | | | | | | | **8** | - | - |
| | | | | | | | | **9** | - | - |
| | | | | | | | | **10** | - | - |
| | | | | | | | | **11** | - | - |
| | | | | | | | | **12** | - | - |
| | | | | | | | | **13** | - | - |
| | | | | | | | | **14** | - | - |
| | | | | | | | | **15** | - | - |
| | | | | | | | | **16** | - | - |
| | | | | | | | | **17** | - | - |
| | | | | | | | | **18** | - | - |
| | | | | | | | | **19** | - | - |
| | | | | | | | | **20** | - | - |

| No of respondent | Profession | Knowledge of the AGREE II instrument | Performance of appraisals using the AGREE I or II instrument | Number of appraised guidelines using the AGREE I or II instrument | Experience in years | Involvement in guideline development | Purpose of conducting appraisals using the AGREE I or II instrument | AGREE II Items | Assessment of the influence on methodological quality | Assessment of the influence on recommendation for use |
|---|---|---|---|---|---|---|---|---|---|---|
| | | | | | | | | **21** | - | - |
| | | | | | | | | **22** | - | - |
| | | | | | | | | **23** | - | - |
| **12** | Physician / methodological expert | yes | yes | < 10 guidelines | 1-5 years | yes | - | **1** | 1 | 1 |
| | | | | | | | | **2** | 2 | 2 |
| | | | | | | | | **3** | 3 | 4 |
| | | | | | | | | **4** | 3 | 2 |
| | | | | | | | | **5** | 4 | 5 |
| | | | | | | | | **6** | 1 | 1 |
| | | | | | | | | **7** | 5 | 5 |
| | | | | | | | | **8** | 5 | 5 |
| | | | | | | | | **9** | 3 | 3 |
| | | | | | | | | **10** | 5 | 5 |
| | | | | | | | | **11** | 5 | 5 |
| | | | | | | | | **12** | 5 | 5 |
| | | | | | | | | **13** | 4 | 2 |
| | | | | | | | | **14** | 3 | 2 |
| | | | | | | | | **15** | 5 | 5 |
| | | | | | | | | **16** | 5 | 5 |
| | | | | | | | | **17** | 2 | 3 |
| | | | | | | | | **18** | 1 | 2 |

| No of respondent | Profession | Knowledge of the AGREE II instrument | Performance of appraisals using the AGREE I or II instrument | Number of appraised guidelines using the AGREE I or II instrument | Experience in years | Involvement in guideline development | Purpose of conducting appraisals using the AGREE I or II instrument | AGREE II Items | Assessment of the influence on methodological quality | Assessment of the influence on recommendation for use |
|---|---|---|---|---|---|---|---|---|---|---|
| | | | | | | | | **19** | 1 | 2 |
| | | | | | | | | **20** | 3 | 4 |
| | | | | | | | | **21** | 1 | 1 |
| | | | | | | | | **22** | 5 | 5 |
| | | | | | | | | **23** | 5 | 5 |
| **13**[b] | other | no | no | - | - | yes | - | **1** | - | - |
| | | | | | | | | **2** | - | - |
| | | | | | | | | **3** | - | - |
| | | | | | | | | **4** | - | - |
| | | | | | | | | **5** | - | - |
| | | | | | | | | **6** | - | - |
| | | | | | | | | **7** | - | - |
| | | | | | | | | **8** | - | - |
| | | | | | | | | **9** | - | - |
| | | | | | | | | **10** | - | - |
| | | | | | | | | **11** | - | - |
| | | | | | | | | **12** | - | - |
| | | | | | | | | **13** | - | - |
| | | | | | | | | **14** | - | - |
| | | | | | | | | **15** | - | - |
| | | | | | | | | **16** | - | - |

| No of respondent | Profession | Knowledge of the AGREE II instrument | Performance of appraisals using the AGREE I or II instrument | Number of appraised guidelines using the AGREE I or II instrument | Experience in years | Involvement in guideline development | Purpose of conducting appraisals using the AGREE I or II instrument | AGREE II Items | Assessment of the influence on methodological quality | Assessment of the influence on recommendation for use |
|---|---|---|---|---|---|---|---|---|---|---|
| | | | | | | | | **17** | - | - |
| | | | | | | | | **18** | - | - |
| | | | | | | | | **19** | - | - |
| | | | | | | | | **20** | - | - |
| | | | | | | | | **21** | - | - |
| | | | | | | | | **22** | - | - |
| | | | | | | | | **23** | - | - |
| **14**[b] | Physician | no | no | - | - | no | - | **1** | - | - |
| | | | | | | | | **2** | - | - |
| | | | | | | | | **3** | - | - |
| | | | | | | | | **4** | - | - |
| | | | | | | | | **5** | - | - |
| | | | | | | | | **6** | - | - |
| | | | | | | | | **7** | - | - |
| | | | | | | | | **8** | - | - |
| | | | | | | | | **9** | - | - |
| | | | | | | | | **10** | - | - |
| | | | | | | | | **11** | - | - |
| | | | | | | | | **12** | - | - |
| | | | | | | | | **13** | - | - |
| | | | | | | | | **14** | - | - |

| **No of respondent** | **Profession** | **Knowledge of the AGREE II instrument** | **Performance of appraisals using the AGREE I or II instrument** | **Number of appraised guidelines using the AGREE I or II instrument** | **Experience in years** | **Involvement in guideline development** | **Purpose of conducting appraisals using the AGREE I or II instrument** | **AGREE II Items** | **Assessment of the influence on methodological quality** | **Assessment of the influence on recommendation for use** |
|---|---|---|---|---|---|---|---|---|---|---|
| | | | | | | | | **15** | - | - |
| | | | | | | | | **16** | - | - |
| | | | | | | | | **17** | - | - |
| | | | | | | | | **18** | - | - |
| | | | | | | | | **19** | - | - |
| | | | | | | | | **20** | - | - |
| | | | | | | | | **21** | - | - |
| | | | | | | | | **22** | - | - |
| | | | | | | | | **23** | - | - |
| **15** | Physician / methodological expert | yes | yes | > 20 guidelines | 1-5 years | yes | Assessment of guideline quality, Development of guidelines | **1** | 1 | 1 |
| | | | | | | | | **2** | 1 | 2 |
| | | | | | | | | **3** | 2 | 2 |
| | | | | | | | | **4** | 3 | 3 |
| | | | | | | | | **5** | 3 | 2 |
| | | | | | | | | **6** | 0 | 0 |
| | | | | | | | | **7** | 5 | 4 |
| | | | | | | | | **8** | 5 | 4 |
| | | | | | | | | **9** | 5 | 4 |
| | | | | | | | | **10** | 4 | 3 |
| | | | | | | | | **11** | 5 | 5 |
| | | | | | | | | **12** | 5 | 3 |

| No of respondent | Profession | Knowledge of the AGREE II instrument | Performance of appraisals using the AGREE I or II instrument | Number of appraised guidelines using the AGREE I or II instrument | Experience in years | Involvement in guideline development | Purpose of conducting appraisals using the AGREE I or II instrument | AGREE II Items | Assessment of the influence on methodological quality | Assessment of the influence on recommendation for use |
|---|---|---|---|---|---|---|---|---|---|---|
| | | | | | | | | **13** | 4 | 3 |
| | | | | | | | | **14** | 1 | 0 |
| | | | | | | | | **15** | 3 | 4 |
| | | | | | | | | **16** | 3 | 4 |
| | | | | | | | | **17** | 4 | 5 |
| | | | | | | | | **18** | 2 | 1 |
| | | | | | | | | **19** | 3 | 4 |
| | | | | | | | | **20** | 4 | 2 |
| | | | | | | | | **21** | 3 | 4 |
| | | | | | | | | **22** | 4 | 4 |
| | | | | | | | | **23** | 4 | 3 |
| **16** | Physician | yes | yes | < 10 guidelines | 1-5 years | no | Further training | **1** | 1 | 4 |
| | | | | | | | | **2** | 2 | 4 |
| | | | | | | | | **3** | 1 | 4 |
| | | | | | | | | **4** | 2 | 3 |
| | | | | | | | | **5** | 3 | 4 |
| | | | | | | | | **6** | 2 | 4 |
| | | | | | | | | **7** | 5 | 4 |
| | | | | | | | | **8** | 5 | 4 |
| | | | | | | | | **9** | 5 | 4 |
| | | | | | | | | **10** | 5 | 4 |

| No of respondent | Profession | Knowledge of the AGREE II instrument | Performance of appraisals using the AGREE I or II instrument | Number of appraised guidelines using the AGREE I or II instrument | Experience in years | Involvement in guideline development | Purpose of conducting appraisals using the AGREE I or II instrument | AGREE II Items | Assessment of the influence on methodological quality | Assessment of the influence on recommendation for use |
|---|---|---|---|---|---|---|---|---|---|---|
| | | | | | | | | **11** | 5 | 4 |
| | | | | | | | | **12** | 5 | 4 |
| | | | | | | | | **13** | 4 | 4 |
| | | | | | | | | **14** | 4 | 1 |
| | | | | | | | | **15** | 2 | 3 |
| | | | | | | | | **16** | 4 | 3 |
| | | | | | | | | **17** | 1 | 4 |
| | | | | | | | | **18** | 3 | 3 |
| | | | | | | | | **19** | 2 | 3 |
| | | | | | | | | **20** | 2 | 3 |
| | | | | | | | | **21** | 4 | 2 |
| | | | | | | | | **22** | 4 | 4 |
| | | | | | | | | **23** | 5 | 3 |
| **17** | methodological expert | yes | yes | > 20 guidelines | > 5 years | no | Assessment of guideline quality | **1** | 1 | 3 |
| | | | | | | | | **2** | 2 | 3 |
| | | | | | | | | **3** | 2 | 4 |
| | | | | | | | | **4** | 3 | 3 |
| | | | | | | | | **5** | 3 | 4 |
| | | | | | | | | **6** | 3 | 4 |
| | | | | | | | | **7** | 5 | 4 |
| | | | | | | | | **8** | 5 | 4 |

| No of respondent | Profession | Knowledge of the AGREE II instrument | Performance of appraisals using the AGREE I or II instrument | Number of appraised guidelines using the AGREE I or II instrument | Experience in years | Involvement in guideline development | Purpose of conducting appraisals using the AGREE I or II instrument | AGREE II Items | Assessment of the influence on methodological quality | Assessment of the influence on recommendation for use |
|---|---|---|---|---|---|---|---|---|---|---|
| | | | | | | | | **9** | 5 | 4 |
| | | | | | | | | **10** | 5 | 4 |
| | | | | | | | | **11** | 4 | 4 |
| | | | | | | | | **12** | 5 | 4 |
| | | | | | | | | **13** | 4 | 4 |
| | | | | | | | | **14** | 2 | 2 |
| | | | | | | | | **15** | 4 | 5 |
| | | | | | | | | **16** | 4 | 4 |
| | | | | | | | | **17** | 3 | 5 |
| | | | | | | | | **18** | 2 | 3 |
| | | | | | | | | **19** | 1 | 3 |
| | | | | | | | | **20** | 2 | 3 |
| | | | | | | | | **21** | 3 | 4 |
| | | | | | | | | **22** | 5 | 3 |
| | | | | | | | | **23** | 5 | 3 |
| **18**[b] | Physician | no | no | - | - | yes | - | **1** | 4 | 4 |
| | | | | | | | | **2** | 4 | 4 |
| | | | | | | | | **3** | 4 | 4 |
| | | | | | | | | **4** | 3 | 3 |
| | | | | | | | | **5** | 3 | 3 |
| | | | | | | | | **6** | 4 | 4 |

| No of respondent | Profession | Knowledge of the AGREE II instrument | Performance of appraisals using the AGREE I or II instrument | Number of appraised guidelines using the AGREE I or II instrument | Experience in years | Involvement in guideline development | Purpose of conducting appraisals using the AGREE I or II instrument | AGREE II Items | Assessment of the influence on methodological quality | Assessment of the influence on recommendation for use |
|---|---|---|---|---|---|---|---|---|---|---|
| | | | | | | | | **7** | 4 | 4 |
| | | | | | | | | **8** | 4 | 4 |
| | | | | | | | | **9** | 3 | 3 |
| | | | | | | | | **10** | 3 | 3 |
| | | | | | | | | **11** | 4 | 4 |
| | | | | | | | | **12** | 3 | 3 |
| | | | | | | | | **13** | 4 | 4 |
| | | | | | | | | **14** | 4 | 4 |
| | | | | | | | | **15** | 4 | 4 |
| | | | | | | | | **16** | 4 | 4 |
| | | | | | | | | **17** | 4 | 4 |
| | | | | | | | | **18** | 3 | 3 |
| | | | | | | | | **19** | 4 | 4 |
| | | | | | | | | **20** | 3 | 3 |
| | | | | | | | | **21** | 4 | 4 |
| | | | | | | | | **22** | 3 | 3 |
| | | | | | | | | **23** | 4 | 4 |
| **19**[a] | other | yes | no | - | - | yes | - | **1** | - | - |
| | | | | | | | | **2** | - | - |
| | | | | | | | | **3** | - | - |
| | | | | | | | | **4** | - | - |

| No of respondent | Profession | Knowledge of the AGREE II instrument | Performance of appraisals using the AGREE I or II instrument | Number of appraised guidelines using the AGREE I or II instrument | Experience in years | Involvement in guideline development | Purpose of conducting appraisals using the AGREE I or II instrument | AGREE II Items | Assessment of the influence on methodological quality | Assessment of the influence on recommendation for use |
|---|---|---|---|---|---|---|---|---|---|---|
| | | | | | | | | **5** | - | - |
| | | | | | | | | **6** | - | - |
| | | | | | | | | **7** | - | - |
| | | | | | | | | **8** | - | - |
| | | | | | | | | **9** | - | - |
| | | | | | | | | **10** | - | - |
| | | | | | | | | **11** | - | - |
| | | | | | | | | **12** | - | - |
| | | | | | | | | **13** | - | - |
| | | | | | | | | **14** | - | - |
| | | | | | | | | **15** | - | - |
| | | | | | | | | **16** | - | - |
| | | | | | | | | **17** | - | - |
| | | | | | | | | **18** | - | - |
| | | | | | | | | **19** | - | - |
| | | | | | | | | **20** | - | - |
| | | | | | | | | **21** | - | - |
| | | | | | | | | **22** | - | - |
| | | | | | | | | **23** | - | - |
| **20**[a] | methodological expert | yes | yes | > 20 guidelines | 1-5 years | no | Writing of guideline | **1** | - | - |
| | | | | | | | | **2** | - | - |

| **No of respondent** | **Profession** | **Knowledge of the AGREE II instrument** | **Performance of appraisals using the AGREE I or II instrument** | **Number of appraised guidelines using the AGREE I or II instrument** | **Experience in years** | **Involvement in guideline development** | **Purpose of conducting appraisals using the AGREE I or II instrument** | **AGREE II Items** | **Assessment of the influence on <u>methodological quality</u>** | **Assessment of the influence on <u>recommendation for use</u>** |
|---|---|---|---|---|---|---|---|---|---|---|
| | | | | | | | synopses | **3** | - | - |
| | | | | | | | | **4** | - | - |
| | | | | | | | | **5** | - | - |
| | | | | | | | | **6** | - | - |
| | | | | | | | | **7** | - | - |
| | | | | | | | | **8** | - | - |
| | | | | | | | | **9** | - | - |
| | | | | | | | | **10** | - | - |
| | | | | | | | | **11** | - | - |
| | | | | | | | | **12** | - | - |
| | | | | | | | | **13** | - | - |
| | | | | | | | | **14** | - | - |
| | | | | | | | | **15** | - | - |
| | | | | | | | | **16** | - | - |
| | | | | | | | | **17** | - | - |
| | | | | | | | | **18** | - | - |
| | | | | | | | | **19** | - | - |
| | | | | | | | | **20** | - | - |
| | | | | | | | | **21** | - | - |
| | | | | | | | | **22** | - | - |
| | | | | | | | | **23** | - | - |

| **No of respondent** | **Profession** | **Knowledge of the AGREE II instrument** | **Performance of appraisals using the AGREE I or II instrument** | **Number of appraised guidelines using the AGREE I or II instrument** | **Experience in years** | **Involvement in guideline development** | **Purpose of conducting appraisals using the AGREE I or II instrument** | **AGREE II Items** | **Assessment of the influence on <u>methodological quality</u>** | **Assessment of the influence on <u>recommendation for use</u>** |
|---|---|---|---|---|---|---|---|---|---|---|
| **21** | Physician | yes | yes | < 10 guidelines | 1-5 years | yes | Development of guidelines, writing of guideline synopses | **1** | 2 | 2 |
| | | | | | | | | **2** | 4 | 4 |
| | | | | | | | | **3** | 5 | 5 |
| | | | | | | | | **4** | 4 | 4 |
| | | | | | | | | **5** | 4 | 4 |
| | | | | | | | | **6** | 5 | 5 |
| | | | | | | | | **7** | 4 | 4 |
| | | | | | | | | **8** | 5 | 5 |
| | | | | | | | | **9** | 5 | 5 |
| | | | | | | | | **10** | 5 | 5 |
| | | | | | | | | **11** | 5 | 5 |
| | | | | | | | | **12** | 4 | 4 |
| | | | | | | | | **13** | 4 | 4 |
| | | | | | | | | **14** | 4 | 4 |
| | | | | | | | | **15** | 4 | 5 |
| | | | | | | | | **16** | 4 | 5 |
| | | | | | | | | **17** | 4 | 5 |
| | | | | | | | | **18** | 3 | 4 |
| | | | | | | | | **19** | 3 | 4 |
| | | | | | | | | **20** | 4 | 5 |
| | | | | | | | | **21** | 4 | 3 |

| **No of respondent** | **Profession** | **Knowledge of the AGREE II instrument** | **Performance of appraisals using the AGREE I or II instrument** | **Number of appraised guidelines using the AGREE I or II instrument** | **Experience in years** | **Involvement in guideline development** | **Purpose of conducting appraisals using the AGREE I or II instrument** | **AGREE II Items** | **Assessment of the influence on methodological quality** | **Assessment of the influence on recommendation for use** |
|---|---|---|---|---|---|---|---|---|---|---|
| | | | | | | | | **22** | 5 | 4 |
| | | | | | | | | **23** | 5 | 3 |
| **22**[b] | Physician | no | no | - | - | no | - | **1** | 0 | 0 |
| | | | | | | | | **2** | 3 | 5 |
| | | | | | | | | **3** | 3 | 5 |
| | | | | | | | | **4** | 5 | 5 |
| | | | | | | | | **5** | 5 | 5 |
| | | | | | | | | **6** | 2 | 5 |
| | | | | | | | | **7** | 5 | 5 |
| | | | | | | | | **8** | 5 | 5 |
| | | | | | | | | **9** | 5 | 5 |
| | | | | | | | | **10** | 5 | 2 |
| | | | | | | | | **11** | 5 | 5 |
| | | | | | | | | **12** | 5 | 5 |
| | | | | | | | | **13** | 5 | 3 |
| | | | | | | | | **14** | 3 | 1 |
| | | | | | | | | **15** | 5 | 5 |
| | | | | | | | | **16** | 5 | 5 |
| | | | | | | | | **17** | 3 | 3 |
| | | | | | | | | **18** | 1 | 1 |
| | | | | | | | | **19** | 1 | 1 |

| No of respondent | Profession | Knowledge of the AGREE II instrument | Performance of appraisals using the AGREE I or II instrument | Number of appraised guidelines using the AGREE I or II instrument | Experience in years | Involvement in guideline development | Purpose of conducting appraisals using the AGREE I or II instrument | AGREE II Items | Assessment of the influence on <u>methodological quality</u> | Assessment of the influence on <u>recommendation for use</u> |
|---|---|---|---|---|---|---|---|---|---|---|
| | | | | | | | | **20** | 1 | 5 |
| | | | | | | | | **21** | 5 | 5 |
| | | | | | | | | **22** | 5 | 5 |
| | | | | | | | | **23** | 5 | 5 |
| **23** | Physician / methodological expert | yes | yes | > 20 guidelines | > 5 years | yes | Writing of guideline synopses, Assessment of guideline quality | **1** | 2 | 3 |
| | | | | | | | | **2** | 2 | 3 |
| | | | | | | | | **3** | 2 | 3 |
| | | | | | | | | **4** | 1 | 2 |
| | | | | | | | | **5** | 1 | 3 |
| | | | | | | | | **6** | 1 | 3 |
| | | | | | | | | **7** | 5 | 5 |
| | | | | | | | | **8** | 5 | 5 |
| | | | | | | | | **9** | 5 | 5 |
| | | | | | | | | **10** | 5 | 5 |
| | | | | | | | | **11** | 5 | 5 |
| | | | | | | | | **12** | 5 | 5 |
| | | | | | | | | **13** | 3 | 3 |
| | | | | | | | | **14** | 1 | 1 |
| | | | | | | | | **15** | 4 | 4 |
| | | | | | | | | **16** | 4 | 4 |
| | | | | | | | | **17** | 4 | 4 |

| No of respondent | Profession | Knowledge of the AGREE II instrument | Performance of appraisals using the AGREE I or II instrument | Number of appraised guidelines using the AGREE I or II instrument | Experience in years | Involvement in guideline development | Purpose of conducting appraisals using the AGREE I or II instrument | AGREE II Items | Assessment of the influence on methodological quality | Assessment of the influence on recommendation for use |
|---|---|---|---|---|---|---|---|---|---|---|
| | | | | | | | | **18** | 1 | 3 |
| | | | | | | | | **19** | 1 | 3 |
| | | | | | | | | **20** | 1 | 3 |
| | | | | | | | | **21** | 3 | 3 |
| | | | | | | | | **22** | 5 | 5 |
| | | | | | | | | **23** | 5 | 5 |
| **24** | Physician / methodological expert | yes | yes | 10-20 guidelines | 1-5 years | yes | Adaptation of guidelines | **1** | 1 | 2 |
| | | | | | | | | **2** | 1 | 2 |
| | | | | | | | | **3** | 2 | 2 |
| | | | | | | | | **4** | 3 | 4 |
| | | | | | | | | **5** | 3 | 4 |
| | | | | | | | | **6** | 1 | 1 |
| | | | | | | | | **7** | 5 | 4 |
| | | | | | | | | **8** | 5 | 4 |
| | | | | | | | | **9** | 5 | 5 |
| | | | | | | | | **10** | 4 | 5 |
| | | | | | | | | **11** | 5 | 5 |
| | | | | | | | | **12** | 5 | 4 |
| | | | | | | | | **13** | 3 | 2 |
| | | | | | | | | **14** | 3 | 3 |
| | | | | | | | | **15** | 3 | 4 |

| No of respondent | Profession | Knowledge of the AGREE II instrument | Performance of appraisals using the AGREE I or II instrument | Number of appraised guidelines using the AGREE I or II instrument | Experience in years | Involvement in guideline development | Purpose of conducting appraisals using the AGREE I or II instrument | AGREE II Items | Assessment of the influence on methodological quality | Assessment of the influence on recommendation for use |
|---|---|---|---|---|---|---|---|---|---|---|
| | | | | | | | | **16** | 3 | 4 |
| | | | | | | | | **17** | 3 | 3 |
| | | | | | | | | **18** | 3 | 4 |
| | | | | | | | | **19** | 3 | 4 |
| | | | | | | | | **20** | 3 | 4 |
| | | | | | | | | **21** | 3 | 4 |
| | | | | | | | | **22** | 4 | 5 |
| | | | | | | | | **23** | 4 | 5 |
| **25** | methodological expert | yes | yes | 10-20 guidelines | 1-5 years | yes | Application in clinical practice | **1** | 5 | 5 |
| | | | | | | | | **2** | 4 | 4 |
| | | | | | | | | **3** | 5 | 5 |
| | | | | | | | | **4** | 3 | 1 |
| | | | | | | | | **5** | 3 | 1 |
| | | | | | | | | **6** | 5 | 5 |
| | | | | | | | | **7** | 5 | 5 |
| | | | | | | | | **8** | 5 | 5 |
| | | | | | | | | **9** | 5 | 5 |
| | | | | | | | | **10** | 5 | 5 |
| | | | | | | | | **11** | 5 | 5 |
| | | | | | | | | **12** | 5 | 5 |
| | | | | | | | | **13** | 4 | 2 |

| No of respondent | Profession | Knowledge of the AGREE II instrument | Performance of appraisals using the AGREE I or II instrument | Number of appraised guidelines using the AGREE I or II instrument | Experience in years | Involvement in guideline development | Purpose of conducting appraisals using the AGREE I or II instrument | AGREE II Items | Assessment of the influence on methodological quality | Assessment of the influence on recommendation for use |
|---|---|---|---|---|---|---|---|---|---|---|
| | | | | | | | | **14** | 2 | 2 |
| | | | | | | | | **15** | 5 | 5 |
| | | | | | | | | **16** | 4 | 4 |
| | | | | | | | | **17** | 5 | 5 |
| | | | | | | | | **18** | 4 | 2 |
| | | | | | | | | **19** | 3 | 2 |
| | | | | | | | | **20** | 3 | 2 |
| | | | | | | | | **21** | 2 | 2 |
| | | | | | | | | **22** | 5 | 5 |
| | | | | | | | | **23** | 5 | 5 |
| **26** | Physician | yes | yes | < 10 guidelines | < 1 year | no | Assessment of guideline quality | **1** | 4 | 4 |
| | | | | | | | | **2** | 4 | 4 |
| | | | | | | | | **3** | 4 | 4 |
| | | | | | | | | **4** | 4 | 5 |
| | | | | | | | | **5** | 2 | 2 |
| | | | | | | | | **6** | 4 | 4 |
| | | | | | | | | **7** | 5 | 5 |
| | | | | | | | | **8** | 5 | 5 |
| | | | | | | | | **9** | 5 | 5 |
| | | | | | | | | **10** | 5 | 5 |
| | | | | | | | | **11** | 5 | 5 |

| No of respondent | Profession | Knowledge of the AGREE II instrument | Performance of appraisals using the AGREE I or II instrument | Number of appraised guidelines using the AGREE I or II instrument | Experience in years | Involvement in guideline development | Purpose of conducting appraisals using the AGREE I or II instrument | AGREE II Items | Assessment of the influence on methodological quality | Assessment of the influence on recommendation for use |
|---|---|---|---|---|---|---|---|---|---|---|
| | | | | | | | | **12** | 5 | 5 |
| | | | | | | | | **13** | 4 | 5 |
| | | | | | | | | **14** | 5 | 5 |
| | | | | | | | | **15** | 4 | 5 |
| | | | | | | | | **16** | 4 | 5 |
| | | | | | | | | **17** | 5 | 5 |
| | | | | | | | | **18** | 5 | 4 |
| | | | | | | | | **19** | 4 | 5 |
| | | | | | | | | **20** | 4 | 5 |
| | | | | | | | | **21** | 4 | 4 |
| | | | | | | | | **22** | 4 | 4 |
| | | | | | | | | **23** | 5 | 4 |
| **27** | Physician | yes | yes | 10-20 guidelines | 1-5 years | no | Assessment of guideline quality | **1** | 1 | 4 |
| | | | | | | | | **2** | 1 | 4 |
| | | | | | | | | **3** | 1 | 5 |
| | | | | | | | | **4** | 5 | 5 |
| | | | | | | | | **5** | 1 | 1 |
| | | | | | | | | **6** | 2 | 4 |
| | | | | | | | | **7** | 5 | 5 |
| | | | | | | | | **8** | 4 | 4 |
| | | | | | | | | **9** | 5 | 5 |

| **No of respondent** | **Profession** | **Knowledge of the AGREE II instrument** | **Performance of appraisals using the AGREE I or II instrument** | **Number of appraised guidelines using the AGREE I or II instrument** | **Experience in years** | **Involvement in guideline development** | **Purpose of conducting appraisals using the AGREE I or II instrument** | **AGREE II Items** | **Assessment of the influence on <u>methodological quality</u>** | **Assessment of the influence on <u>recommendation for use</u>** |
|---|---|---|---|---|---|---|---|---|---|---|
| | | | | | | | | **10** | 4 | 3 |
| | | | | | | | | **11** | 5 | 5 |
| | | | | | | | | **12** | 5 | 5 |
| | | | | | | | | **13** | 5 | 5 |
| | | | | | | | | **14** | 4 | 4 |
| | | | | | | | | **15** | 4 | 5 |
| | | | | | | | | **16** | 4 | 5 |
| | | | | | | | | **17** | 3 | 5 |
| | | | | | | | | **18** | 3 | 3 |
| | | | | | | | | **19** | 4 | 5 |
| | | | | | | | | **20** | 4 | 5 |
| | | | | | | | | **21** | 3 | 4 |
| | | | | | | | | **22** | 5 | 5 |
| | | | | | | | | **23** | 5 | 5 |
| **28** | other | yes | yes | < 10 guidelines | 1-5 years | yes | Assessment of guideline quality, Updating of guidelines | **1** | 4 | 4 |
| | | | | | | | | **2** | 3 | 4 |
| | | | | | | | | **3** | 3 | 4 |
| | | | | | | | | **4** | 3 | 3 |
| | | | | | | | | **5** | 1 | 5 |
| | | | | | | | | **6** | 2 | 4 |
| | | | | | | | | **7** | 3 | 5 |

| No of respondent | Profession | Knowledge of the AGREE II instrument | Performance of appraisals using the AGREE I or II instrument | Number of appraised guidelines using the AGREE I or II instrument | Experience in years | Involvement in guideline development | Purpose of conducting appraisals using the AGREE I or II instrument | AGREE II Items | Assessment of the influence on methodological quality | Assessment of the influence on recommendation for use |
|---|---|---|---|---|---|---|---|---|---|---|
| | | | | | | | | **8** | 2 | 5 |
| | | | | | | | | **9** | 2 | 5 |
| | | | | | | | | **10** | 4 | 5 |
| | | | | | | | | **11** | 2 | 5 |
| | | | | | | | | **12** | 2 | 5 |
| | | | | | | | | **13** | 4 | 5 |
| | | | | | | | | **14** | 3 | 4 |
| | | | | | | | | **15** | 4 | 5 |
| | | | | | | | | **16** | 3 | 5 |
| | | | | | | | | **17** | 4 | 4 |
| | | | | | | | | **18** | 1 | 4 |
| | | | | | | | | **19** | 1 | 4 |
| | | | | | | | | **20** | 1 | 4 |
| | | | | | | | | **21** | 1 | 4 |
| | | | | | | | | **22** | 4 | 5 |
| | | | | | | | | **23** | 4 | 5 |
| **29** | other | yes | yes | > 20 guidelines | 1-5 years | no | Assessment of guideline quality | **1** | 4 | 4 |
| | | | | | | | | **2** | 4 | 4 |
| | | | | | | | | **3** | 5 | 5 |
| | | | | | | | | **4** | 4 | 4 |
| | | | | | | | | **5** | 4 | 4 |

| No of respondent | Profession | Knowledge of the AGREE II instrument | Performance of appraisals using the AGREE I or II instrument | Number of appraised guidelines using the AGREE I or II instrument | Experience in years | Involvement in guideline development | Purpose of conducting appraisals using the AGREE I or II instrument | AGREE II Items | Assessment of the influence on methodological quality | Assessment of the influence on recommendation for use |
|---|---|---|---|---|---|---|---|---|---|---|
| | | | | | | | | **6** | 4 | 5 |
| | | | | | | | | **7** | 5 | 5 |
| | | | | | | | | **8** | 5 | 5 |
| | | | | | | | | **9** | 4 | 4 |
| | | | | | | | | **10** | 4 | 4 |
| | | | | | | | | **11** | 5 | 5 |
| | | | | | | | | **12** | 5 | 5 |
| | | | | | | | | **13** | 5 | 5 |
| | | | | | | | | **14** | 4 | 3 |
| | | | | | | | | **15** | 5 | 5 |
| | | | | | | | | **16** | 5 | 5 |
| | | | | | | | | **17** | 5 | 5 |
| | | | | | | | | **18** | 4 | 4 |
| | | | | | | | | **19** | 4 | 5 |
| | | | | | | | | **20** | 3 | 4 |
| | | | | | | | | **21** | 4 | 3 |
| | | | | | | | | **22** | 5 | 4 |
| | | | | | | | | **23** | 5 | 4 |
| **30** | Physician / methodological expert | yes | yes | < 10 guidelines | 1-5 years | yes | Research | **1** | 5 | 5 |
| | | | | | | | | **2** | 5 | 5 |
| | | | | | | | | **3** | 5 | 5 |

| No of respondent | Profession | Knowledge of the AGREE II instrument | Performance of appraisals using the AGREE I or II instrument | Number of appraised guidelines using the AGREE I or II instrument | Experience in years | Involvement in guideline development | Purpose of conducting appraisals using the AGREE I or II instrument | AGREE II Items | Assessment of the influence on methodological quality | Assessment of the influence on recommendation for use |
|---|---|---|---|---|---|---|---|---|---|---|
| | | | | | | | | **4** | 3 | 3 |
| | | | | | | | | **5** | 4 | 4 |
| | | | | | | | | **6** | 4 | 4 |
| | | | | | | | | **7** | 5 | 5 |
| | | | | | | | | **8** | 5 | 5 |
| | | | | | | | | **9** | 5 | 5 |
| | | | | | | | | **10** | 5 | 5 |
| | | | | | | | | **11** | 5 | 5 |
| | | | | | | | | **12** | 5 | 5 |
| | | | | | | | | **13** | 5 | 5 |
| | | | | | | | | **14** | 5 | 5 |
| | | | | | | | | **15** | 3 | 3 |
| | | | | | | | | **16** | 3 | 3 |
| | | | | | | | | **17** | 3 | 3 |
| | | | | | | | | **18** | 3 | 3 |
| | | | | | | | | **19** | 3 | 3 |
| | | | | | | | | **20** | 3 | 3 |
| | | | | | | | | **21** | 3 | 3 |
| | | | | | | | | **22** | 5 | 5 |
| | | | | | | | | **23** | 5 | 5 |
| **31** | Physician | yes | yes | < 10 | 1-5 years | no | Research | **1** | 4 | 4 |

| **No of respondent** | **Profession** | **Knowledge of the AGREE II instrument** | **Performance of appraisals using the AGREE I or II instrument** | **Number of appraised guidelines using the AGREE I or II instrument** | **Experience in years** | **Involvement in guideline development** | **Purpose of conducting appraisals using the AGREE I or II instrument** | **AGREE II Items** | **Assessment of the influence on <u>methodological quality</u>** | **Assessment of the influence on <u>recommendation for use</u>** |
|---|---|---|---|---|---|---|---|---|---|---|
| | | | | guidelines | | | | **2** | 4 | 4 |
| | | | | | | | | **3** | 4 | 4 |
| | | | | | | | | **4** | 4 | 4 |
| | | | | | | | | **5** | 4 | 4 |
| | | | | | | | | **6** | 4 | 4 |
| | | | | | | | | **7** | 4 | 4 |
| | | | | | | | | **8** | 4 | 4 |
| | | | | | | | | **9** | 3 | 3 |
| | | | | | | | | **10** | 4 | 3 |
| | | | | | | | | **11** | 3 | 4 |
| | | | | | | | | **12** | 3 | 4 |
| | | | | | | | | **13** | 3 | 4 |
| | | | | | | | | **14** | 4 | 3 |
| | | | | | | | | **15** | 5 | 5 |
| | | | | | | | | **16** | 5 | 5 |
| | | | | | | | | **17** | 5 | 5 |
| | | | | | | | | **18** | 3 | 4 |
| | | | | | | | | **19** | 4 | 4 |
| | | | | | | | | **20** | 3 | 3 |
| | | | | | | | | **21** | 3 | 3 |
| | | | | | | | | **22** | 5 | 5 |

| No of respondent | Profession | Knowledge of the AGREE II instrument | Performance of appraisals using the AGREE I or II instrument | Number of appraised guidelines using the AGREE I or II instrument | Experience in years | Involvement in guideline development | Purpose of conducting appraisals using the AGREE I or II instrument | AGREE II Items | Assessment of the influence on methodological quality | Assessment of the influence on recommendation for use |
|---|---|---|---|---|---|---|---|---|---|---|
| | | | | | | | | **23** | 5 | 5 |
| **32** | Physician | yes | yes | < 10 guidelines | 1-5 years | yes | Assessment of guideline quality, Development of guidelines | **1** | 5 | 5 |
| | | | | | | | | **2** | 5 | 5 |
| | | | | | | | | **3** | 5 | 5 |
| | | | | | | | | **4** | 5 | 5 |
| | | | | | | | | **5** | 4 | 4 |
| | | | | | | | | **6** | 5 | 5 |
| | | | | | | | | **7** | 5 | 5 |
| | | | | | | | | **8** | 5 | 5 |
| | | | | | | | | **9** | 5 | 5 |
| | | | | | | | | **10** | 5 | 5 |
| | | | | | | | | **11** | 4 | 5 |
| | | | | | | | | **12** | 4 | 4 |
| | | | | | | | | **13** | 3 | 3 |
| | | | | | | | | **14** | 5 | 5 |
| | | | | | | | | **15** | 5 | 5 |
| | | | | | | | | **16** | 4 | 5 |
| | | | | | | | | **17** | 5 | 5 |
| | | | | | | | | **18** | 4 | 4 |
| | | | | | | | | **19** | 4 | 4 |
| | | | | | | | | **20** | 4 | 4 |

| No of respondent | Profession | Knowledge of the AGREE II instrument | Performance of appraisals using the AGREE I or II instrument | Number of appraised guidelines using the AGREE I or II instrument | Experience in years | Involvement in guideline development | Purpose of conducting appraisals using the AGREE I or II instrument | AGREE II Items | Assessment of the influence on methodological quality | Assessment of the influence on recommendation for use |
|---|---|---|---|---|---|---|---|---|---|---|
| | | | | | | | | **21** | 4 | 4 |
| | | | | | | | | **22** | 5 | 5 |
| | | | | | | | | **23** | 5 | 5 |
| **33** | Physician | yes | yes | < 10 guidelines | 1-5 years | yes | Application in clinical practice | **1** | 4 | 5 |
| | | | | | | | | **2** | 5 | 5 |
| | | | | | | | | **3** | 5 | 5 |
| | | | | | | | | **4** | 3 | 1 |
| | | | | | | | | **5** | 3 | 1 |
| | | | | | | | | **6** | 4 | 3 |
| | | | | | | | | **7** | 5 | 5 |
| | | | | | | | | **8** | 5 | 5 |
| | | | | | | | | **9** | 4 | 4 |
| | | | | | | | | **10** | 4 | 4 |
| | | | | | | | | **11** | 2 | 4 |
| | | | | | | | | **12** | 4 | 4 |
| | | | | | | | | **13** | 5 | 1 |
| | | | | | | | | **14** | 3 | 0 |
| | | | | | | | | **15** | 4 | 5 |
| | | | | | | | | **16** | 1 | 4 |
| | | | | | | | | **17** | 1 | 5 |
| | | | | | | | | **18** | 1 | 3 |

| No of respondent | Profession | Knowledge of the AGREE II instrument | Performance of appraisals using the AGREE I or II instrument | Number of appraised guidelines using the AGREE I or II instrument | Experience in years | Involvement in guideline development | Purpose of conducting appraisals using the AGREE I or II instrument | AGREE II Items | Assessment of the influence on methodological quality | Assessment of the influence on recommendation for use |
|---|---|---|---|---|---|---|---|---|---|---|
| | | | | | | | | **19** | 1 | 3 |
| | | | | | | | | **20** | 1 | 3 |
| | | | | | | | | **21** | 1 | 3 |
| | | | | | | | | **22** | 5 | 3 |
| | | | | | | | | **23** | 5 | 5 |
| **34** | other | yes | yes | < 10 guidelines | 1-5 years | yes | Development of guidelines | **1** | 5 | 5 |
| | | | | | | | | **2** | 5 | 5 |
| | | | | | | | | **3** | 5 | 5 |
| | | | | | | | | **4** | 5 | 5 |
| | | | | | | | | **5** | 4 | 4 |
| | | | | | | | | **6** | 5 | 5 |
| | | | | | | | | **7** | 5 | 5 |
| | | | | | | | | **8** | 5 | 5 |
| | | | | | | | | **9** | 5 | 5 |
| | | | | | | | | **10** | 5 | 5 |
| | | | | | | | | **11** | 5 | 5 |
| | | | | | | | | **12** | 5 | 5 |
| | | | | | | | | **13** | 5 | 5 |
| | | | | | | | | **14** | 4 | 4 |
| | | | | | | | | **15** | 4 | 4 |
| | | | | | | | | **16** | 4 | 4 |

| No of respondent | Profession | Knowledge of the AGREE II instrument | Performance of appraisals using the AGREE I or II instrument | Number of appraised guidelines using the AGREE I or II instrument | Experience in years | Involvement in guideline development | Purpose of conducting appraisals using the AGREE I or II instrument | AGREE II Items | Assessment of the influence on methodological quality | Assessment of the influence on recommendation for use |
|---|---|---|---|---|---|---|---|---|---|---|
| | | | | | | | | **17** | 5 | 5 |
| | | | | | | | | **18** | 3 | 4 |
| | | | | | | | | **19** | 3 | 4 |
| | | | | | | | | **20** | 3 | 4 |
| | | | | | | | | **21** | 4 | 4 |
| | | | | | | | | **22** | 5 | 5 |
| | | | | | | | | **23** | 5 | 5 |
| **35** | Physician | yes | yes | > 20 guidelines | 1-5 years | no | Assessment of guideline quality | **1** | 4 | 3 |
| | | | | | | | | **2** | 4 | 4 |
| | | | | | | | | **3** | 4 | 4 |
| | | | | | | | | **4** | 4 | 4 |
| | | | | | | | | **5** | 4 | 3 |
| | | | | | | | | **6** | 4 | 4 |
| | | | | | | | | **7** | 4 | 4 |
| | | | | | | | | **8** | 4 | 4 |
| | | | | | | | | **9** | 4 | 4 |
| | | | | | | | | **10** | 4 | 4 |
| | | | | | | | | **11** | 5 | 5 |
| | | | | | | | | **12** | 5 | 5 |
| | | | | | | | | **13** | 5 | 5 |
| | | | | | | | | **14** | 4 | 4 |

| **No of respondent** | **Profession** | **Knowledge of the AGREE II instrument** | **Performance of appraisals using the AGREE I or II instrument** | **Number of appraised guidelines using the AGREE I or II instrument** | **Experience in years** | **Involvement in guideline development** | **Purpose of conducting appraisals using the AGREE I or II instrument** | **AGREE II Items** | **Assessment of the influence on methodological quality** | **Assessment of the influence on recommendation for use** |
|---|---|---|---|---|---|---|---|---|---|---|
| | | | | | | | | **15** | 5 | 5 |
| | | | | | | | | **16** | 4 | 4 |
| | | | | | | | | **17** | 5 | 5 |
| | | | | | | | | **18** | 5 | 5 |
| | | | | | | | | **19** | 5 | 5 |
| | | | | | | | | **20** | 5 | 5 |
| | | | | | | | | **21** | 5 | 5 |
| | | | | | | | | **22** | 5 | 5 |
| | | | | | | | | **23** | 5 | 5 |
| **36** | other | yes | yes | > 20 guidelines | 1-5 years | no | Assessment of guideline quality | **1** | 4 | 3 |
| | | | | | | | | **2** | 3 | 4 |
| | | | | | | | | **3** | 2 | 4 |
| | | | | | | | | **4** | 2 | 3 |
| | | | | | | | | **5** | 2 | 3 |
| | | | | | | | | **6** | 4 | 4 |
| | | | | | | | | **7** | 5 | 5 |
| | | | | | | | | **8** | 5 | 4 |
| | | | | | | | | **9** | 4 | 5 |
| | | | | | | | | **10** | 5 | 5 |
| | | | | | | | | **11** | 4 | 5 |
| | | | | | | | | **12** | 4 | 5 |

| No of respondent | Profession | Knowledge of the AGREE II instrument | Performance of appraisals using the AGREE I or II instrument | Number of appraised guidelines using the AGREE I or II instrument | Experience in years | Involvement in guideline development | Purpose of conducting appraisals using the AGREE I or II instrument | AGREE II Items | Assessment of the influence on methodological quality | Assessment of the influence on recommendation for use |
|---|---|---|---|---|---|---|---|---|---|---|
| | | | | | | | | **13** | 5 | 5 |
| | | | | | | | | **14** | 3 | 5 |
| | | | | | | | | **15** | 4 | 5 |
| | | | | | | | | **16** | 4 | 5 |
| | | | | | | | | **17** | 3 | 5 |
| | | | | | | | | **18** | 3 | 4 |
| | | | | | | | | **19** | 4 | 5 |
| | | | | | | | | **20** | 3 | 4 |
| | | | | | | | | **21** | 4 | 4 |
| | | | | | | | | **22** | 4 | 4 |
| | | | | | | | | **23** | 5 | 4 |
| **37** | Physician | yes | yes | > 20 guidelines | 1-5 years | no | Writing of guideline synopses | **1** | 3 | 3 |
| | | | | | | | | **2** | 2 | 2 |
| | | | | | | | | **3** | 3 | 3 |
| | | | | | | | | **4** | 4 | 4 |
| | | | | | | | | **5** | 4 | 4 |
| | | | | | | | | **6** | 3 | 3 |
| | | | | | | | | **7** | 5 | 5 |
| | | | | | | | | **8** | 5 | 5 |
| | | | | | | | | **9** | 4 | 4 |
| | | | | | | | | **10** | 5 | 5 |

| **No of respondent** | **Profession** | **Knowledge of the AGREE II instrument** | **Performance of appraisals using the AGREE I or II instrument** | **Number of appraised guidelines using the AGREE I or II instrument** | **Experience in years** | **Involvement in guideline development** | **Purpose of conducting appraisals using the AGREE I or II instrument** | **AGREE II Items** | **Assessment of the influence on methodological quality** | **Assessment of the influence on recommendation for use** |
|---|---|---|---|---|---|---|---|---|---|---|
| | | | | | | | | **11** | 5 | 5 |
| | | | | | | | | **12** | 5 | 5 |
| | | | | | | | | **13** | 4 | 4 |
| | | | | | | | | **14** | 3 | 3 |
| | | | | | | | | **15** | 4 | 4 |
| | | | | | | | | **16** | 4 | 4 |
| | | | | | | | | **17** | 3 | 3 |
| | | | | | | | | **18** | 2 | 2 |
| | | | | | | | | **19** | 1 | 1 |
| | | | | | | | | **20** | 1 | 1 |
| | | | | | | | | **21** | 0 | 0 |
| | | | | | | | | **22** | 4 | 4 |
| | | | | | | | | **23** | 4 | 4 |
| **38** | other | yes | yes | < 10 guidelines | 1-5 years | yes | Assessment of guideline quality, Development of knowledge tools | **1** | 2 | 5 |
| | | | | | | | | **2** | 2 | 5 |
| | | | | | | | | **3** | 4 | 5 |
| | | | | | | | | **4** | 4 | 4 |
| | | | | | | | | **5** | 4 | 4 |
| | | | | | | | | **6** | 4 | 4 |
| | | | | | | | | **7** | 5 | 5 |
| | | | | | | | | **8** | 5 | 5 |

| No of respondent | Profession | Knowledge of the AGREE II instrument | Performance of appraisals using the AGREE I or II instrument | Number of appraised guidelines using the AGREE I or II instrument | Experience in years | Involvement in guideline development | Purpose of conducting appraisals using the AGREE I or II instrument | AGREE II Items | Assessment of the influence on methodological quality | Assessment of the influence on recommendation for use |
|---|---|---|---|---|---|---|---|---|---|---|
| | | | | | | | | **9** | 5 | 5 |
| | | | | | | | | **10** | 5 | 5 |
| | | | | | | | | **11** | 4 | 4 |
| | | | | | | | | **12** | 5 | 5 |
| | | | | | | | | **13** | 5 | 5 |
| | | | | | | | | **14** | 4 | 4 |
| | | | | | | | | **15** | 4 | 4 |
| | | | | | | | | **16** | 5 | 5 |
| | | | | | | | | **17** | 4 | 4 |
| | | | | | | | | **18** | 3 | 3 |
| | | | | | | | | **19** | 5 | 5 |
| | | | | | | | | **20** | 4 | 4 |
| | | | | | | | | **21** | 4 | 4 |
| | | | | | | | | **22** | 5 | 5 |
| | | | | | | | | **23** | 5 | 5 |
| **39** | methodological expert | yes | yes | 10-20 guidelines | > 5 years | yes | further training | **1** | 2 | 3 |
| | | | | | | | | **2** | 4 | 3 |
| | | | | | | | | **3** | 4 | 4 |
| | | | | | | | | **4** | 4 | 4 |
| | | | | | | | | **5** | 4 | 3 |
| | | | | | | | | **6** | 3 | 2 |

| No of respondent | Profession | Knowledge of the AGREE II instrument | Performance of appraisals using the AGREE I or II instrument | Number of appraised guidelines using the AGREE I or II instrument | Experience in years | Involvement in guideline development | Purpose of conducting appraisals using the AGREE I or II instrument | AGREE II Items | Assessment of the influence on methodological quality | Assessment of the influence on recommendation for use |
|---|---|---|---|---|---|---|---|---|---|---|
| | | | | | | | | **7** | 5 | 5 |
| | | | | | | | | **8** | 3 | 2 |
| | | | | | | | | **9** | 5 | 5 |
| | | | | | | | | **10** | 5 | 5 |
| | | | | | | | | **11** | 5 | 5 |
| | | | | | | | | **12** | 4 | 4 |
| | | | | | | | | **13** | 4 | 2 |
| | | | | | | | | **14** | 3 | 1 |
| | | | | | | | | **15** | 4 | 4 |
| | | | | | | | | **16** | 3 | 5 |
| | | | | | | | | **17** | 4 | 5 |
| | | | | | | | | **18** | 4 | 4 |
| | | | | | | | | **19** | 3 | 4 |
| | | | | | | | | **20** | 4 | 4 |
| | | | | | | | | **21** | 3 | 3 |
| | | | | | | | | **22** | 3 | 2 |
| | | | | | | | | **23** | 5 | 5 |
| **40** | Physician | yes | yes | < 10 guidelines | < 1 year | no | Assessment of guideline quality | **1** | 5 | 5 |
| | | | | | | | | **2** | 3 | 5 |
| | | | | | | | | **3** | 4 | 5 |
| | | | | | | | | **4** | 5 | 5 |

| **No of respondent** | **Profession** | **Knowledge of the AGREE II instrument** | **Performance of appraisals using the AGREE I or II instrument** | **Number of appraised guidelines using the AGREE I or II instrument** | **Experience in years** | **Involvement in guideline development** | **Purpose of conducting appraisals using the AGREE I or II instrument** | **AGREE II Items** | **Assessment of the influence on methodological quality** | **Assessment of the influence on recommendation for use** |
|---|---|---|---|---|---|---|---|---|---|---|
| | | | | | | | | **5** | 3 | 5 |
| | | | | | | | | **6** | 4 | 5 |
| | | | | | | | | **7** | 5 | 5 |
| | | | | | | | | **8** | 5 | 5 |
| | | | | | | | | **9** | 5 | 4 |
| | | | | | | | | **10** | 5 | 3 |
| | | | | | | | | **11** | 5 | 5 |
| | | | | | | | | **12** | 5 | 5 |
| | | | | | | | | **13** | 5 | 3 |
| | | | | | | | | **14** | 4 | 4 |
| | | | | | | | | **15** | 2 | 2 |
| | | | | | | | | **16** | 4 | 5 |
| | | | | | | | | **17** | 5 | 5 |
| | | | | | | | | **18** | 1 | 3 |
| | | | | | | | | **19** | 2 | 3 |
| | | | | | | | | **20** | 4 | 5 |
| | | | | | | | | **21** | 1 | 4 |
| | | | | | | | | **22** | 5 | 5 |
| | | | | | | | | **23** | 5 | 5 |
| **41** | other | yes | yes | < 10 guidelines | 1-5 years | no | Assessment of guideline | **1** | 3 | 3 |
| | | | | | | | | **2** | 3 | 3 |

| No of respondent | Profession | Knowledge of the AGREE II instrument | Performance of appraisals using the AGREE I or II instrument | Number of appraised guidelines using the AGREE I or II instrument | Experience in years | Involvement in guideline development | Purpose of conducting appraisals using the AGREE I or II instrument | AGREE II Items | Assessment of the influence on methodological quality | Assessment of the influence on recommendation for use |
|---|---|---|---|---|---|---|---|---|---|---|
| | | | | | | | quality | **3** | 3 | 3 |
| | | | | | | | | **4** | 4 | 3 |
| | | | | | | | | **5** | 4 | 3 |
| | | | | | | | | **6** | 4 | 2 |
| | | | | | | | | **7** | 5 | 5 |
| | | | | | | | | **8** | 5 | 5 |
| | | | | | | | | **9** | 5 | 4 |
| | | | | | | | | **10** | 5 | 4 |
| | | | | | | | | **11** | 5 | 5 |
| | | | | | | | | **12** | 5 | 5 |
| | | | | | | | | **13** | 5 | 5 |
| | | | | | | | | **14** | 4 | 3 |
| | | | | | | | | **15** | 5 | 5 |
| | | | | | | | | **16** | 5 | 5 |
| | | | | | | | | **17** | 5 | 5 |
| | | | | | | | | **18** | 5 | 4 |
| | | | | | | | | **19** | 5 | 4 |
| | | | | | | | | **20** | 5 | 4 |
| | | | | | | | | **21** | 4 | 3 |
| | | | | | | | | **22** | 5 | 5 |
| | | | | | | | | **23** | 5 | 5 |

| No of respondent | Profession | Knowledge of the AGREE II instrument | Performance of appraisals using the AGREE I or II instrument | Number of appraised guidelines using the AGREE I or II instrument | Experience in years | Involvement in guideline development | Purpose of conducting appraisals using the AGREE I or II instrument | AGREE II Items | Assessment of the influence on methodological quality | Assessment of the influence on recommendation for use |
|---|---|---|---|---|---|---|---|---|---|---|
| **42** | Physician | yes | yes | < 10 guidelines | < 1 year | no | Writing of guideline synopses | **1** | 4 | 4 |
| | | | | | | | | **2** | 3 | 4 |
| | | | | | | | | **3** | 3 | 4 |
| | | | | | | | | **4** | 4 | 3 |
| | | | | | | | | **5** | 3 | 3 |
| | | | | | | | | **6** | 4 | 4 |
| | | | | | | | | **7** | 5 | 4 |
| | | | | | | | | **8** | 5 | 4 |
| | | | | | | | | **9** | 5 | 5 |
| | | | | | | | | **10** | 5 | 5 |
| | | | | | | | | **11** | 4 | 5 |
| | | | | | | | | **12** | 5 | 5 |
| | | | | | | | | **13** | 4 | 4 |
| | | | | | | | | **14** | 3 | 4 |
| | | | | | | | | **15** | 4 | 5 |
| | | | | | | | | **16** | 5 | 5 |
| | | | | | | | | **17** | 5 | 5 |
| | | | | | | | | **18** | 3 | 4 |
| | | | | | | | | **19** | 3 | 3 |
| | | | | | | | | **20** | 3 | 3 |
| | | | | | | | | **21** | 3 | 3 |

| No of respondent | Profession | Knowledge of the AGREE II instrument | Performance of appraisals using the AGREE I or II instrument | Number of appraised guidelines using the AGREE I or II instrument | Experience in years | Involvement in guideline development | Purpose of conducting appraisals using the AGREE I or II instrument | AGREE II Items | Assessment of the influence on methodological quality | Assessment of the influence on recommendation for use |
|---|---|---|---|---|---|---|---|---|---|---|
| | | | | | | | | **22** | 5 | 5 |
| | | | | | | | | **23** | 5 | 4 |
| **43** | Physician / methodological expert | yes | yes | > 20 guidelines | > 5 years | yes | Adaptation of guidelines | **1** | 1 | 1 |
| | | | | | | | | **2** | 5 | 5 |
| | | | | | | | | **3** | 4 | 4 |
| | | | | | | | | **4** | 5 | 3 |
| | | | | | | | | **5** | 4 | 3 |
| | | | | | | | | **6** | 3 | 2 |
| | | | | | | | | **7** | 5 | 5 |
| | | | | | | | | **8** | 5 | 4 |
| | | | | | | | | **9** | 5 | 4 |
| | | | | | | | | **10** | 4 | 3 |
| | | | | | | | | **11** | 5 | 5 |
| | | | | | | | | **12** | 5 | 5 |
| | | | | | | | | **13** | 3 | 2 |
| | | | | | | | | **14** | 2 | 0 |
| | | | | | | | | **15** | 2 | 4 |
| | | | | | | | | **16** | 2 | 3 |
| | | | | | | | | **17** | 0 | 2 |
| | | | | | | | | **18** | 1 | 2 |
| | | | | | | | | **19** | 0 | 2 |

| **No of respondent** | **Profession** | **Knowledge of the AGREE II instrument** | **Performance of appraisals using the AGREE I or II instrument** | **Number of appraised guidelines using the AGREE I or II instrument** | **Experience in years** | **Involvement in guideline development** | **Purpose of conducting appraisals using the AGREE I or II instrument** | **AGREE II Items** | **Assessment of the influence on <u>methodological quality</u>** | **Assessment of the influence on <u>recommendation for use</u>** |
|---|---|---|---|---|---|---|---|---|---|---|
| | | | | | | | | **20** | 1 | 1 |
| | | | | | | | | **21** | 1 | 0 |
| | | | | | | | | **22** | 5 | 5 |
| | | | | | | | | **23** | 5 | 5 |
| **44** | Physician | yes | yes | < 10 guidelines | 1-5 years | yes | Assessment of guideline quality | **1** | 5 | 5 |
| | | | | | | | | **2** | 5 | 5 |
| | | | | | | | | **3** | 5 | 5 |
| | | | | | | | | **4** | 2 | 3 |
| | | | | | | | | **5** | 3 | 3 |
| | | | | | | | | **6** | 3 | 5 |
| | | | | | | | | **7** | 5 | 3 |
| | | | | | | | | **8** | 5 | 4 |
| | | | | | | | | **9** | 5 | 4 |
| | | | | | | | | **10** | 5 | 5 |
| | | | | | | | | **11** | 3 | 4 |
| | | | | | | | | **12** | 5 | 4 |
| | | | | | | | | **13** | 4 | 4 |
| | | | | | | | | **14** | 2 | 2 |
| | | | | | | | | **15** | 5 | 5 |
| | | | | | | | | **16** | 4 | 4 |
| | | | | | | | | **17** | 5 | 5 |

| No of respondent | Profession | Knowledge of the AGREE II instrument | Performance of appraisals using the AGREE I or II instrument | Number of appraised guidelines using the AGREE I or II instrument | Experience in years | Involvement in guideline development | Purpose of conducting appraisals using the AGREE I or II instrument | AGREE II Items | Assessment of the influence on methodological quality | Assessment of the influence on recommendation for use |
|---|---|---|---|---|---|---|---|---|---|---|
| | | | | | | | | **18** | 3 | 3 |
| | | | | | | | | **19** | 4 | 4 |
| | | | | | | | | **20** | 3 | 5 |
| | | | | | | | | **21** | 3 | 4 |
| | | | | | | | | **22** | 5 | 5 |
| | | | | | | | | **23** | 5 | 4 |
| **45** | methodological expert | yes | yes | 10-20 guidelines | 1-5 years | yes | Assessment of guideline quality, Writing of guideline synopses | **1** | 2 | 2 |
| | | | | | | | | **2** | 3 | 2 |
| | | | | | | | | **3** | 3 | 3 |
| | | | | | | | | **4** | 2 | 2 |
| | | | | | | | | **5** | 2 | 2 |
| | | | | | | | | **6** | 2 | 3 |
| | | | | | | | | **7** | 5 | 3 |
| | | | | | | | | **8** | 4 | 2 |
| | | | | | | | | **9** | 3 | 2 |
| | | | | | | | | **10** | 3 | 2 |
| | | | | | | | | **11** | 3 | 3 |
| | | | | | | | | **12** | 5 | 5 |
| | | | | | | | | **13** | 3 | 3 |
| | | | | | | | | **14** | 1 | 1 |
| | | | | | | | | **15** | 3 | 4 |

| **No of respondent** | **Profession** | **Knowledge of the AGREE II instrument** | **Performance of appraisals using the AGREE I or II instrument** | **Number of appraised guidelines using the AGREE I or II instrument** | **Experience in years** | **Involvement in guideline development** | **Purpose of conducting appraisals using the AGREE I or II instrument** | **AGREE II Items** | **Assessment of the influence on methodological quality** | **Assessment of the influence on recommendation for use** |
|---|---|---|---|---|---|---|---|---|---|---|
| | | | | | | | | **16** | 3 | 4 |
| | | | | | | | | **17** | 3 | 4 |
| | | | | | | | | **18** | 4 | 3 |
| | | | | | | | | **19** | 4 | 4 |
| | | | | | | | | **20** | 3 | 3 |
| | | | | | | | | **21** | 3 | 3 |
| | | | | | | | | **22** | 4 | 4 |
| | | | | | | | | **23** | 2 | 2 |
| **46** | Physician / methodological expert | yes | yes | 10-20 guidelines | > 5 years | yes | - | **1** | 5 | 5 |
| | | | | | | | | **2** | 5 | 5 |
| | | | | | | | | **3** | 5 | 5 |
| | | | | | | | | **4** | 4 | 4 |
| | | | | | | | | **5** | 3 | 3 |
| | | | | | | | | **6** | 4 | 4 |
| | | | | | | | | **7** | 5 | 5 |
| | | | | | | | | **8** | 4 | 4 |
| | | | | | | | | **9** | 4 | 4 |
| | | | | | | | | **10** | 5 | 5 |
| | | | | | | | | **11** | 4 | 4 |
| | | | | | | | | **12** | 5 | 5 |
| | | | | | | | | **13** | 4 | 4 |

| No of respondent | Profession | Knowledge of the AGREE II instrument | Performance of appraisals using the AGREE I or II instrument | Number of appraised guidelines using the AGREE I or II instrument | Experience in years | Involvement in guideline development | Purpose of conducting appraisals using the AGREE I or II instrument | AGREE II Items | Assessment of the influence on methodological quality | Assessment of the influence on recommendation for use |
|---|---|---|---|---|---|---|---|---|---|---|
| | | | | | | | | **14** | 3 | 3 |
| | | | | | | | | **15** | 5 | 5 |
| | | | | | | | | **16** | 1 | 1 |
| | | | | | | | | **17** | 5 | 5 |
| | | | | | | | | **18** | 1 | 1 |
| | | | | | | | | **19** | 1 | 1 |
| | | | | | | | | **20** | 3 | 3 |
| | | | | | | | | **21** | 1 | 1 |
| | | | | | | | | **22** | 5 | 5 |
| | | | | | | | | **23** | 5 | 5 |
| **47** | other | yes | yes | > 20 guidelines | > 5 years | yes | Assessment of guideline quality | **1** | 4 | 4 |
| | | | | | | | | **2** | 5 | 4 |
| | | | | | | | | **3** | 5 | 4 |
| | | | | | | | | **4** | 5 | 5 |
| | | | | | | | | **5** | 5 | 5 |
| | | | | | | | | **6** | 5 | 5 |
| | | | | | | | | **7** | 5 | 4 |
| | | | | | | | | **8** | 5 | 4 |
| | | | | | | | | **9** | 5 | 5 |
| | | | | | | | | **10** | 5 | 5 |
| | | | | | | | | **11** | 5 | 5 |

| **No of respondent** | **Profession** | **Knowledge of the AGREE II instrument** | **Performance of appraisals using the AGREE I or II instrument** | **Number of appraised guidelines using the AGREE I or II instrument** | **Experience in years** | **Involvement in guideline development** | **Purpose of conducting appraisals using the AGREE I or II instrument** | **AGREE II Items** | **Assessment of the influence on <u>methodological quality</u>** | **Assessment of the influence on <u>recommendation for use</u>** |
|---|---|---|---|---|---|---|---|---|---|---|
| | | | | | | | | **12** | 5 | 5 |
| | | | | | | | | **13** | 5 | 4 |
| | | | | | | | | **14** | 4 | 4 |
| | | | | | | | | **15** | 5 | 5 |
| | | | | | | | | **16** | 5 | 5 |
| | | | | | | | | **17** | 4 | 5 |
| | | | | | | | | **18** | 4 | 5 |
| | | | | | | | | **19** | 4 | 5 |
| | | | | | | | | **20** | 4 | 5 |
| | | | | | | | | **21** | 4 | 5 |
| | | | | | | | | **22** | 4 | 4 |
| | | | | | | | | **23** | 4 | 4 |
| **48** | Physician | yes | yes | < 10 guidelines | < 1 year | no | Assessment of guideline quality | **1** | 3 | 3 |
| | | | | | | | | **2** | 3 | 4 |
| | | | | | | | | **3** | 3 | 4 |
| | | | | | | | | **4** | 4 | 3 |
| | | | | | | | | **5** | 4 | 3 |
| | | | | | | | | **6** | 4 | 3 |
| | | | | | | | | **7** | 5 | 5 |
| | | | | | | | | **8** | 5 | 5 |
| | | | | | | | | **9** | 4 | 4 |

| No of respondent | Profession | Knowledge of the AGREE II instrument | Performance of appraisals using the AGREE I or II instrument | Number of appraised guidelines using the AGREE I or II instrument | Experience in years | Involvement in guideline development | Purpose of conducting appraisals using the AGREE I or II instrument | AGREE II Items | Assessment of the influence on methodological quality | Assessment of the influence on recommendation for use |
|---|---|---|---|---|---|---|---|---|---|---|
| | | | | | | | | **10** | 4 | 4 |
| | | | | | | | | **11** | 4 | 4 |
| | | | | | | | | **12** | 5 | 4 |
| | | | | | | | | **13** | 5 | 5 |
| | | | | | | | | **14** | 4 | 2 |
| | | | | | | | | **15** | 4 | 5 |
| | | | | | | | | **16** | 4 | 4 |
| | | | | | | | | **17** | 4 | 5 |
| | | | | | | | | **18** | 4 | 2 |
| | | | | | | | | **19** | 4 | 5 |
| | | | | | | | | **20** | 4 | 2 |
| | | | | | | | | **21** | 4 | 2 |
| | | | | | | | | **22** | 4 | 4 |
| | | | | | | | | **23** | 4 | 3 |
| **49** | methodological expert | yes | yes | 10-20 guidelines | 1-5 years | no | Assessment of guideline quality | **1** | 1 | 2 |
| | | | | | | | | **2** | 1 | 2 |
| | | | | | | | | **3** | 1 | 2 |
| | | | | | | | | **4** | 1 | 1 |
| | | | | | | | | **5** | 1 | 1 |
| | | | | | | | | **6** | 1 | 1 |
| | | | | | | | | **7** | 5 | 5 |

| **No of respondent** | **Profession** | **Knowledge of the AGREE II instrument** | **Performance of appraisals using the AGREE I or II instrument** | **Number of appraised guidelines using the AGREE I or II instrument** | **Experience in years** | **Involvement in guideline development** | **Purpose of conducting appraisals using the AGREE I or II instrument** | **AGREE II Items** | **Assessment of the influence on methodological quality** | **Assessment of the influence on recommendation for use** |
|---|---|---|---|---|---|---|---|---|---|---|
| | | | | | | | | **8** | 4 | 4 |
| | | | | | | | | **9** | 5 | 5 |
| | | | | | | | | **10** | 3 | 3 |
| | | | | | | | | **11** | 5 | 5 |
| | | | | | | | | **12** | 4 | 4 |
| | | | | | | | | **13** | 3 | 3 |
| | | | | | | | | **14** | 1 | 1 |
| | | | | | | | | **15** | 3 | 4 |
| | | | | | | | | **16** | 3 | 3 |
| | | | | | | | | **17** | 3 | 3 |
| | | | | | | | | **18** | 1 | 1 |
| | | | | | | | | **19** | 1 | 1 |
| | | | | | | | | **20** | 1 | 1 |
| | | | | | | | | **21** | 0 | 0 |
| | | | | | | | | **22** | 4 | 4 |
| | | | | | | | | **23** | 4 | 4 |
| **50** | other | yes | yes | < 10 guidelines | < 1 year | yes | Research | **1** | 4 | 4 |
| | | | | | | | | **2** | 4 | 5 |
| | | | | | | | | **3** | 3 | 3 |
| | | | | | | | | **4** | 4 | 4 |
| | | | | | | | | **5** | 3 | 4 |

| No of respondent | Profession | Knowledge of the AGREE II instrument | Performance of appraisals using the AGREE I or II instrument | Number of appraised guidelines using the AGREE I or II instrument | Experience in years | Involvement in guideline development | Purpose of conducting appraisals using the AGREE I or II instrument | AGREE II Items | Assessment of the influence on methodological quality | Assessment of the influence on recommendation for use |
|---|---|---|---|---|---|---|---|---|---|---|
| | | | | | | | | **6** | 4 | 4 |
| | | | | | | | | **7** | 5 | 5 |
| | | | | | | | | **8** | 4 | 4 |
| | | | | | | | | **9** | 5 | 5 |
| | | | | | | | | **10** | 4 | 4 |
| | | | | | | | | **11** | 3 | 3 |
| | | | | | | | | **12** | 4 | 4 |
| | | | | | | | | **13** | 4 | 4 |
| | | | | | | | | **14** | 5 | 5 |
| | | | | | | | | **15** | 4 | 4 |
| | | | | | | | | **16** | 4 | 5 |
| | | | | | | | | **17** | 5 | 5 |
| | | | | | | | | **18** | 4 | 4 |
| | | | | | | | | **19** | 4 | 4 |
| | | | | | | | | **20** | 4 | 5 |
| | | | | | | | | **21** | 4 | 5 |
| | | | | | | | | **22** | 4 | 5 |
| | | | | | | | | **23** | 5 | 5 |
| **51** | Physician | yes | yes | < 10 guidelines | < 1 year | no | Assessment of guideline quality | **1** | 3 | 4 |
| | | | | | | | | **2** | 3 | 3 |
| | | | | | | | | **3** | 3 | 3 |

| No of respondent | Profession | Knowledge of the AGREE II instrument | Performance of appraisals using the AGREE I or II instrument | Number of appraised guidelines using the AGREE I or II instrument | Experience in years | Involvement in guideline development | Purpose of conducting appraisals using the AGREE I or II instrument | AGREE II Items | Assessment of the influence on methodological quality | Assessment of the influence on recommendation for use |
|---|---|---|---|---|---|---|---|---|---|---|
| | | | | | | | | **4** | 5 | 5 |
| | | | | | | | | **5** | 5 | 5 |
| | | | | | | | | **6** | 4 | 4 |
| | | | | | | | | **7** | 5 | 5 |
| | | | | | | | | **8** | 5 | 5 |
| | | | | | | | | **9** | 5 | 5 |
| | | | | | | | | **10** | 5 | 4 |
| | | | | | | | | **11** | 5 | 5 |
| | | | | | | | | **12** | 5 | 5 |
| | | | | | | | | **13** | 4 | 4 |
| | | | | | | | | **14** | 3 | 3 |
| | | | | | | | | **15** | 4 | 4 |
| | | | | | | | | **16** | 4 | 4 |
| | | | | | | | | **17** | 4 | 5 |
| | | | | | | | | **18** | 4 | 4 |
| | | | | | | | | **19** | 3 | 3 |
| | | | | | | | | **20** | 3 | 3 |
| | | | | | | | | **21** | 3 | 3 |
| | | | | | | | | **22** | 5 | 4 |
| | | | | | | | | **23** | 5 | 4 |
| **52** | Physician / | yes | yes | < 10 | 1-5 years | yes | - | **1** | 5 | 3 |

| **No of respondent** | **Profession** | **Knowledge of the AGREE II instrument** | **Performance of appraisals using the AGREE I or II instrument** | **Number of appraised guidelines using the AGREE I or II instrument** | **Experience in years** | **Involvement in guideline development** | **Purpose of conducting appraisals using the AGREE I or II instrument** | **AGREE II Items** | **Assessment of the influence on <u>methodological quality</u>** | **Assessment of the influence on <u>recommendation for use</u>** |
|---|---|---|---|---|---|---|---|---|---|---|
| | methodological expert | | | guidelines | | | | **2** | 3 | 3 |
| | | | | | | | | **3** | 3 | 4 |
| | | | | | | | | **4** | 3 | 2 |
| | | | | | | | | **5** | 2 | 2 |
| | | | | | | | | **6** | 3 | 3 |
| | | | | | | | | **7** | 3 | 3 |
| | | | | | | | | **8** | 5 | 3 |
| | | | | | | | | **9** | 5 | 3 |
| | | | | | | | | **10** | 2 | 2 |
| | | | | | | | | **11** | 4 | 4 |
| | | | | | | | | **12** | 5 | 5 |
| | | | | | | | | **13** | 2 | 2 |
| | | | | | | | | **14** | 1 | 1 |
| | | | | | | | | **15** | 2 | 4 |
| | | | | | | | | **16** | 2 | 4 |
| | | | | | | | | **17** | 3 | 2 |
| | | | | | | | | **18** | 3 | 2 |
| | | | | | | | | **19** | 3 | 3 |
| | | | | | | | | **20** | 2 | 2 |
| | | | | | | | | **21** | 2 | 1 |
| | | | | | | | | **22** | 2 | 1 |

| No of respondent | Profession | Knowledge of the AGREE II instrument | Performance of appraisals using the AGREE I or II instrument | Number of appraised guidelines using the AGREE I or II instrument | Experience in years | Involvement in guideline development | Purpose of conducting appraisals using the AGREE I or II instrument | AGREE II Items | Assessment of the influence on methodological quality | Assessment of the influence on recommendation for use |
|---|---|---|---|---|---|---|---|---|---|---|
| | | | | | | | | **23** | 5 | 3 |
| **53** | other | yes | yes | < 10 guidelines | 1-5 years | no | Assessment of guideline quality | **1** | 4 | 1 |
| | | | | | | | | **2** | 3 | 1 |
| | | | | | | | | **3** | 3 | 4 |
| | | | | | | | | **4** | 5 | 4 |
| | | | | | | | | **5** | 3 | 2 |
| | | | | | | | | **6** | 5 | 5 |
| | | | | | | | | **7** | 5 | 5 |
| | | | | | | | | **8** | 4 | 2 |
| | | | | | | | | **9** | 4 | 2 |
| | | | | | | | | **10** | 4 | 3 |
| | | | | | | | | **11** | 4 | 5 |
| | | | | | | | | **12** | 3 | 4 |
| | | | | | | | | **13** | 5 | 3 |
| | | | | | | | | **14** | 3 | 2 |
| | | | | | | | | **15** | 3 | 5 |
| | | | | | | | | **16** | 3 | 5 |
| | | | | | | | | **17** | 3 | 5 |
| | | | | | | | | **18** | 3 | 3 |
| | | | | | | | | **19** | 3 | 3 |
| | | | | | | | | **20** | 3 | 3 |

| No of respondent | Profession | Knowledge of the AGREE II instrument | Performance of appraisals using the AGREE I or II instrument | Number of appraised guidelines using the AGREE I or II instrument | Experience in years | Involvement in guideline development | Purpose of conducting appraisals using the AGREE I or II instrument | AGREE II Items | Assessment of the influence on methodological quality | Assessment of the influence on recommendation for use |
|---|---|---|---|---|---|---|---|---|---|---|
| | | | | | | | | **21** | 3 | 3 |
| | | | | | | | | **22** | 5 | 5 |
| | | | | | | | | **23** | 5 | 5 |
| **54** | Physician | yes | yes | < 10 guidelines | 1-5 years | yes | Development of guidelines | **1** | 4 | 4 |
| | | | | | | | | **2** | 5 | 5 |
| | | | | | | | | **3** | 5 | 5 |
| | | | | | | | | **4** | 4 | 3 |
| | | | | | | | | **5** | 4 | 3 |
| | | | | | | | | **6** | 4 | 4 |
| | | | | | | | | **7** | 5 | 5 |
| | | | | | | | | **8** | 5 | 5 |
| | | | | | | | | **9** | 5 | 4 |
| | | | | | | | | **10** | 5 | 4 |
| | | | | | | | | **11** | 5 | 5 |
| | | | | | | | | **12** | 5 | 5 |
| | | | | | | | | **13** | 5 | 4 |
| | | | | | | | | **14** | 4 | 4 |
| | | | | | | | | **15** | 4 | 4 |
| | | | | | | | | **16** | 4 | 4 |
| | | | | | | | | **17** | 5 | 5 |
| | | | | | | | | **18** | 4 | 3 |

| No of respondent | Profession | Knowledge of the AGREE II instrument | Performance of appraisals using the AGREE I or II instrument | Number of appraised guidelines using the AGREE I or II instrument | Experience in years | Involvement in guideline development | Purpose of conducting appraisals using the AGREE I or II instrument | AGREE II Items | Assessment of the influence on methodological quality | Assessment of the influence on recommendation for use |
|---|---|---|---|---|---|---|---|---|---|---|
| | | | | | | | | **19** | 3 | 3 |
| | | | | | | | | **20** | 4 | 3 |
| | | | | | | | | **21** | 3 | 3 |
| | | | | | | | | **22** | 5 | 5 |
| | | | | | | | | **23** | 5 | 5 |
| **55**[c] | other | yes | yes | > 20 guidelines | > 5 years | yes | Assessment of guideline quality | **1** | 5 | 3 |
| | | | | | | | | **2** | 5 | 3 |
| | | | | | | | | **3** | 5 | 5 |
| | | | | | | | | **4** | 5 | 5 |
| | | | | | | | | **5** | 5 | 5 |
| | | | | | | | | **6** | 5 | 5 |
| | | | | | | | | **7** | - | - |
| | | | | | | | | **8** | - | - |
| | | | | | | | | **9** | - | - |
| | | | | | | | | **10** | - | - |
| | | | | | | | | **11** | - | - |
| | | | | | | | | **12** | - | - |
| | | | | | | | | **13** | - | - |
| | | | | | | | | **14** | - | - |
| | | | | | | | | **15** | - | - |
| | | | | | | | | **16** | - | - |

| No of respondent | Profession | Knowledge of the AGREE II instrument | Performance of appraisals using the AGREE I or II instrument | Number of appraised guidelines using the AGREE I or II instrument | Experience in years | Involvement in guideline development | Purpose of conducting appraisals using the AGREE I or II instrument | AGREE II Items | Assessment of the influence on methodological quality | Assessment of the influence on recommendation for use |
|---|---|---|---|---|---|---|---|---|---|---|
| | | | | | | | | **17** | - | - |
| | | | | | | | | **18** | - | - |
| | | | | | | | | **19** | - | - |
| | | | | | | | | **20** | - | - |
| | | | | | | | | **21** | - | - |
| | | | | | | | | **22** | - | - |
| | | | | | | | | **23** | - | - |
| **56**[c] | Physician / methodological expert | yes | yes | < 10 guidelines | 1-5 years | yes | Assessment of guideline quality | **1** | 5 | 5 |
| | | | | | | | | **2** | 5 | 5 |
| | | | | | | | | **3** | 5 | 4 |
| | | | | | | | | **4** | 4 | 4 |
| | | | | | | | | **5** | 4 | 4 |
| | | | | | | | | **6** | 4 | 4 |
| | | | | | | | | **7** | 5 | 5 |
| | | | | | | | | **8** | 5 | 5 |
| | | | | | | | | **9** | 4 | 4 |
| | | | | | | | | **10** | 5 | 5 |
| | | | | | | | | **11** | 4 | 5 |
| | | | | | | | | **12** | 5 | 5 |
| | | | | | | | | **13** | 4 | 4 |
| | | | | | | | | **14** | 5 | 5 |

| No of respondent | Profession | Knowledge of the AGREE II instrument | Performance of appraisals using the AGREE I or II instrument | Number of appraised guidelines using the AGREE I or II instrument | Experience in years | Involvement in guideline development | Purpose of conducting appraisals using the AGREE I or II instrument | AGREE II Items | Assessment of the influence on methodological quality | Assessment of the influence on recommendation for use |
|---|---|---|---|---|---|---|---|---|---|---|
| | | | | | | | | **15** | 4 | 5 |
| | | | | | | | | **16** | 4 | 5 |
| | | | | | | | | **17** | 5 | 5 |
| | | | | | | | | **18** | - | - |
| | | | | | | | | **19** | - | - |
| | | | | | | | | **20** | - | - |
| | | | | | | | | **21** | - | - |
| | | | | | | | | **22** | - | - |
| | | | | | | | | **23** | - | - |
| **57** | methodological expert | yes | yes | > 20 guidelines | > 5 years | yes | Assessment of guideline quality | **1** | 4 | 3 |
| | | | | | | | | **2** | 4 | 4 |
| | | | | | | | | **3** | 5 | 5 |
| | | | | | | | | **4** | 5 | 5 |
| | | | | | | | | **5** | 4 | 4 |
| | | | | | | | | **6** | 5 | 5 |
| | | | | | | | | **7** | 5 | 5 |
| | | | | | | | | **8** | 5 | 5 |
| | | | | | | | | **9** | 5 | 5 |
| | | | | | | | | **10** | 5 | 5 |
| | | | | | | | | **11** | 5 | 5 |
| | | | | | | | | **12** | 5 | 5 |

| **No of respondent** | **Profession** | **Knowledge of the AGREE II instrument** | **Performance of appraisals using the AGREE I or II instrument** | **Number of appraised guidelines using the AGREE I or II instrument** | **Experience in years** | **Involvement in guideline development** | **Purpose of conducting appraisals using the AGREE I or II instrument** | **AGREE II Items** | **Assessment of the influence on methodological quality** | **Assessment of the influence on recommendation for use** |
|---|---|---|---|---|---|---|---|---|---|---|
| | | | | | | | | **13** | 5 | 5 |
| | | | | | | | | **14** | 4 | 4 |
| | | | | | | | | **15** | 4 | 4 |
| | | | | | | | | **16** | 4 | 4 |
| | | | | | | | | **17** | 4 | 4 |
| | | | | | | | | **18** | 2 | 2 |
| | | | | | | | | **19** | 2 | 2 |
| | | | | | | | | **20** | 2 | 2 |
| | | | | | | | | **21** | 2 | 2 |
| | | | | | | | | **22** | 5 | 5 |
| | | | | | | | | **23** | 5 | 5 |
| **58** | other | yes | yes | 10-20 guidelines | 1-5 years | no | Publication of scientific articles | **1** | 4 | 4 |
| | | | | | | | | **2** | 4 | 4 |
| | | | | | | | | **3** | 2 | 4 |
| | | | | | | | | **4** | 4 | 4 |
| | | | | | | | | **5** | 4 | 4 |
| | | | | | | | | **6** | 4 | 4 |
| | | | | | | | | **7** | 5 | 5 |
| | | | | | | | | **8** | 5 | 5 |
| | | | | | | | | **9** | 5 | 5 |
| | | | | | | | | **10** | 4 | 4 |

| No of respondent | Profession | Knowledge of the AGREE II instrument | Performance of appraisals using the AGREE I or II instrument | Number of appraised guidelines using the AGREE I or II instrument | Experience in years | Involvement in guideline development | Purpose of conducting appraisals using the AGREE I or II instrument | AGREE II Items | Assessment of the influence on methodological quality | Assessment of the influence on recommendation for use |
|---|---|---|---|---|---|---|---|---|---|---|
| | | | | | | | | **11** | 4 | 4 |
| | | | | | | | | **12** | 5 | 5 |
| | | | | | | | | **13** | 4 | 4 |
| | | | | | | | | **14** | 5 | 5 |
| | | | | | | | | **15** | 5 | 5 |
| | | | | | | | | **16** | 4 | 4 |
| | | | | | | | | **17** | 5 | 5 |
| | | | | | | | | **18** | 4 | 4 |
| | | | | | | | | **19** | 4 | 4 |
| | | | | | | | | **20** | 4 | 4 |
| | | | | | | | | **21** | 4 | 4 |
| | | | | | | | | **22** | 4 | 4 |
| | | | | | | | | **23** | 4 | 4 |

a: These respondents provided no evaluation of the influence of the AGREE II items.

b: These respondents reported that they had no knowledge of AGREE II. Two of them still answer the further questions. These results were excluded from further analysis.

c: These respondents discontinued their evaluation of the influence of the AGREE II items at Item 7 and Item 8.
